# Computation‐Less, Bias‐Free, and Ultra‐Broadband Photodetector for On‐Chip Edge Extraction

**DOI:** 10.1002/adma.74925

**Published:** 2026-09-11

**Authors:** Jintao Fu, Hao Jiang, Xingsi Liu, Fakun Wang, Yang Peng, Qi Jie Wang, Weibo Gao, Xingzhan Wei, Cheng‐Wei Qiu

**Affiliations:** ^1^ Department of Electrical and Computer Engineering National University of Singapore Singapore Singapore; ^2^ School of Electrical and Electronic Engineering Nanyang Technological University Singapore Singapore; ^3^ Chongqing Institute of Green and Intelligent Technology Chinese Academy of Sciences Chongqing P. R. China

**Keywords:** edge extraction, image processing, infrared photodetection, on‐chip, photodetector

## Abstract

Edges represent the critical structural features in visual information, and their extraction is essential for image processing. However, mainstream edge extraction technologies either rely on numerical computation implemented in software or hardware, or require auxiliary delicate optical components, thereby facing challenges in energy consumption, structural complexity, or operational spectral range. Here, a physical‐mechanism‐empowered, edge‐sensitive photodetector is proposed to achieve computation‐less on‐chip edge extraction, featuring bias‐free operation and an ultra‐broadband photoresponse. The device exhibits a position‐dependent bi‐directional photoresponse driven by the diffusion and asymmetrical transport of photocarriers, thereby realizing an intrinsic spatial differentiation. Consequently, a single device can directly perceive both edge intensity and orientation at the pixel level. Experimental results also demonstrate that the device can operate without any external power supply and exhibits an ultra‐broad operating spectral range from the near‐infrared to the long‐wave infrared at room temperature, with commendable sensitivity to blackbody radiation. The results highlight the feasibility of exploiting intrinsic physical mechanisms for edge extraction, offering an insightful pathway toward the development of compact, low‐power, and functional optoelectronic devices.

## Graphical abstract

A computation‐less, bias‐free, and ultra‐broadband photodetector directly converts local optical‐intensity gradients into signed photocurrents through a position‐dependent bi‐directional photoresponse. This intrinsic physical sensing mechanism enables single‐pixel edge extraction from the near‐infrared to the long‐wave infrared at room temperature, offering a compact route toward dedicated on‐chip edge sensing without numerical convolution.

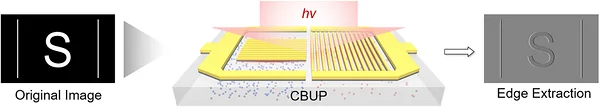

## 1 Introduction

In an image, an edge typically refers to regions where the intensity exhibits an abrupt spatial variation, which may arise from surface discontinuities, reflectance differences, or illumination boundaries [1]. Edges carry crucial information about the structural and morphological characteristics of a scene. Effective edge extraction can reduce the amount of data to be processed, simplify image analysis, and preserve the essential boundary features of objects [2]. Therefore, edge extraction is one of the fundamental tasks in image processing, playing a central role in image segmentation, object recognition, and machine vision [3].

Conventional edge extraction methods mainly rely on numerical computation [4, 5], such as employing Sobel or Laplacian operators to perform 2D convolution. Specifically, the image sensor captures the scene and generates analog electrical signals that are subsequently read out, digitized, and transferred to a central processor, where numerical computation is applied to extract edge features (Figure 1a). However, because the sensing and processing modules are physically separated, the large volume of raw data acquired by the sensor must undergo analog‐to‐digital conversion, storage, and transmission before being processed. Frequent data transfer along the signal chain results in high energy consumption, latency, and hardware overhead [6, 7, 8, 9].

**FIGURE 1:**
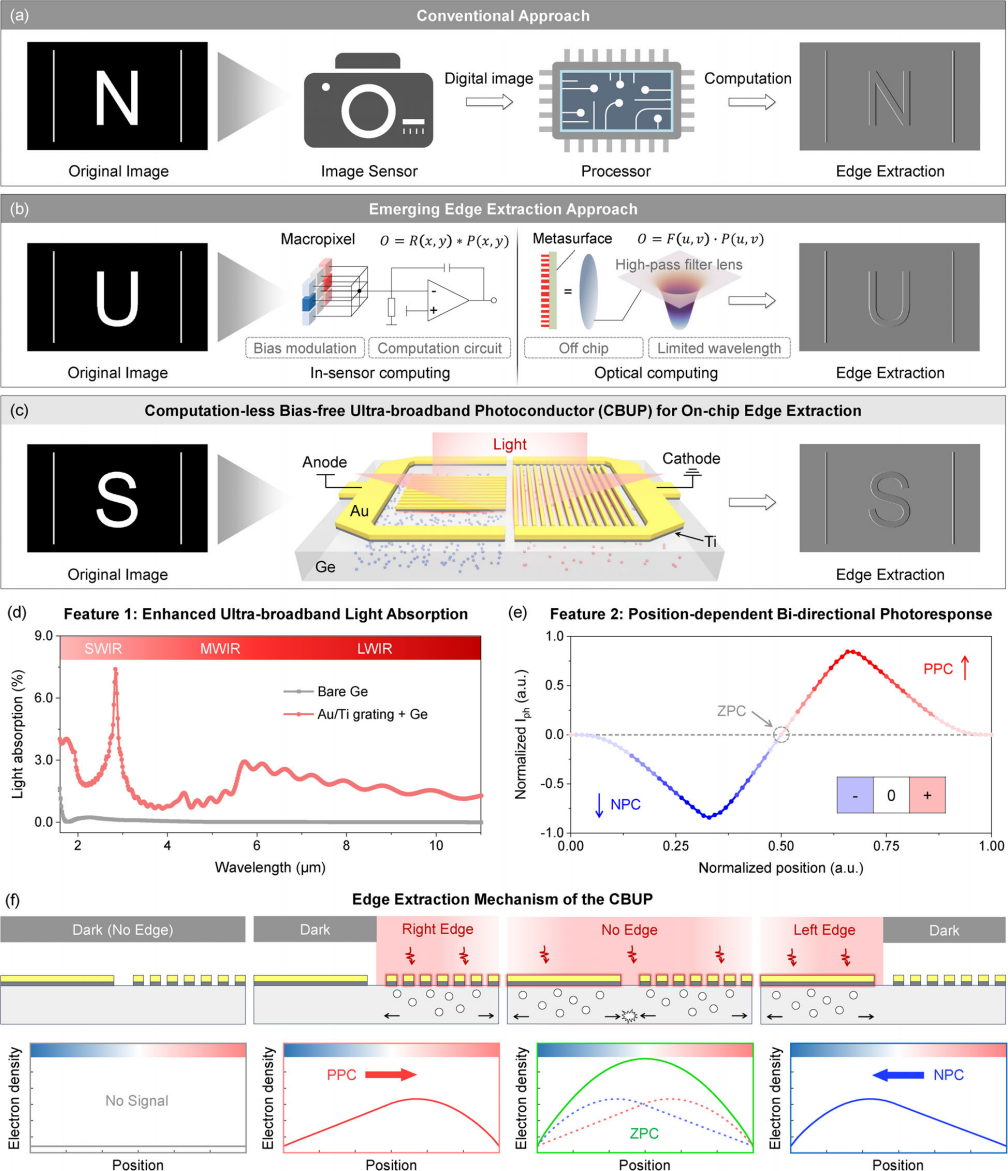
Concept and mechanism of the CBUP. (a) Schematic of conventional edge extraction approach, which relies on physically separated image sensors and processors. (b) Edge extraction based on in‐sensor computing and optical computing. In‐sensor computing employs reconfigurable photodetectors, which are assembled into macro‐pixels together with peripheral circuits, to perform 2D convolution operations at the sensing end. In the inserted equation, *O* represents the output signal, * denotes convolution, *R*(*x*, *y*) is the responsivity of the pixel at position (*x*, *y*), and *P*(*x*, *y*) is the optical power at the same position. Optical computing leverages metasurfaces with high‐pass filtering characteristics. When light passes through the metasurface, it is equivalent to performing multiplication in the frequency domain, as shown in the inserted equation, where *O* denotes the output signal, drepresents multiplication, *F*(*u*, *v*) is the frequency‐domain response of the metasurface, and *P*(*u*, *v*) is the Fourier‐transformed optical power distribution. (c) Edge extraction enabled by the CBUP, and its schematic structure. Red and blue spheres denote photocarriers, with colors only indicating the polarity of the resulting photocurrent. The current flowing out of (into) the anode is defined as positive (negative). Original images with the letters “N”, “U”, and “S”, as well as their edge maps, are obtained from simulations. (d) Simulated light absorption of the CBUP with and without metallic gratings. (e) Simulated position‐dependent bi‐directional photoresponse and the corresponding OEDV symbol. (f) Edge extraction principle of the CBUP. Electron density curves are obtained from simulation corresponding to CBUP with an OEDV of [−1, 0, 1] (Section S6).

To overcome these challenges, the concept of in‐sensor computing and optical computing [10, 11] has been proposed to reduce latency and enhance efficiency (Figure 1b). In‐sensor computing aims to perform numerical computation directly at the sensing front end. To realize in‐sensor computing, various reconfigurable photodetectors with tunable positive and negative photoresponses have been developed [12, 13, 14, 15, 16, 17]. By assembling multiple reconfigurable photodetectors into a macro‐pixel array and precisely tuning their individual photoresponse weights, operators such as Sobel and Laplacian can be constructed. Furthermore, by integrating summation circuits or utilizing Kirchhoff's law to accumulate photocurrents at circuit nodes, convolution operations for edge extraction can be implemented. However, the external voltage and the circuit design necessary for photoresponse weight modulation and photocurrent computation contributes to power consumption and hardware complexity. Optical computing achieves edge extraction by designing metasurfaces or metamaterials that possess spatial high‐pass filtering characteristics [18, 19, 20, 21, 22]. In this approach, the image received by the sensor corresponds to the edge version of the scene. Since the edge extraction process originates from the intrinsic interaction between light and micro/nanostructures, it requires no external voltage supply and therefore offers remarkable advantages in low‐power consumption. Nevertheless, optical components used for optical computing typically function within limited spectral ranges, and the requirement of a propagation distance between the optical component and the detector hinders compact on‐chip integration. Therefore, although recent outstanding advances in edge extraction technologies have demonstrated significant improvements over conventional approaches, achieving a computation‐less, bias‐free, ultra‐broadband, and on‐chip edge detection paradigm remains elusive.

In this work, a physically empowered edge‐sensitivity enhancement strategy is proposed to substitute numerical computation, thereby enabling computation‐less on‐chip edge extraction (Figure 1c). The term *Computation‐less* here indicates the ability of the designed device to perceive edge feature through its intrinsic physical mechanism, without relying on external voltage supplies, complex computing circuit layouts, or auxiliary optical filters. The proposed device, referred to as a computation‐less bias‐free ultra‐broadband photoconductor (CBUP), is derived from a normal photoconductor but incorporates subwavelength metallic gratings with asymmetric spatial layouts in the left and right photosensitive regions. The CBUP exhibits two features. (1) It delivers a bias‐free ultra‐broadband photoresponse, operating from the near‐infrared (1.55 µm) to the long‐wave infrared (11 µm) at room temperature, surpassing the bandgap limitation of the semiconductor channel. (2) It exhibits a position‐dependent bi‐directional photocurrent, where both the polarity and amplitude of the photocurrent vary with the illumination position. This position dependence renders the CBUP inherently sensitive to spatial intensity discontinuities, thereby enabling edge extraction. Experimental results demonstrate that a single CBUP can distinguish between upper/lower and left/right edges without any numerical computation. In contrast, conventional numerical‐operation‐based edge extraction approaches rely on the intensity information of a 3 × 3 pixel array surrounding the target pixel.

## 2 Results and Discussion

### 2.1 Concept and Design

Figure 1c schematically illustrates the structure of the CBUP, in which the germanium (Ge) substrate serves as the channel, and a subwavelength metallic grating is integrated on top. The incorporation of the metallic grating significantly enhances the light absorption of the CBUP from the short‐wave to the long‐wave infrared region (Figure 1d, Section S1), which is essential for achieving ultra‐broadband operation. The design concept of the CBUP is inspired by the mathematical principle of spatial differentiation (Section S2), manifested as a position‐dependent bi‐directional photoresponse that enables computation‐less edge extraction.

The position‐dependent bi‐directional photoresponse refers to the phenomenon that when the incident light spot moves across different regions of the channel, the photocurrent varies not only in magnitude but also in polarity (Figure 1e). This phenomenon is related to the differential diffusion and bipolar transport of photocarriers under zero external bias. Specifically, localized and nonuniform optical excitation generates spatially varying electron and hole concentrations within the Ge channel. Both photogenerated electrons and holes diffuse from regions of high carrier concentration toward regions of low carrier concentration. However, because electron and hole have different diffusion coefficients, their spatial distributions become separated during diffusion, thereby forming a photoinduced Dember electric field. This field further regulates electron and hole transport, allowing the device to reach a nonequilibrium steady state under continuous illumination and to generate a stable photocurrent under short‐circuit conditions. The resulting short‐circuit photocurrent *I_SC_* follows the relation (Section S3),

(1) $$I_{SC}\propto \left(\mu_{n}-\mu_{p}\right)\left(G_{R}-G_{L}\right)$$

where *µ_n_* and *µ_p_* denote the electron and hole mobilities, respectively, and *G_R_*/*G_L_* represent the photocarrier generation rates in the right/left photosensitive regions. Since *µ_n_* > *µ_p_* in Ge, the polarity of the *I_SC_* is determined by the difference between *G_R_* and *G_L_*. Specifically, when the light spot is located in the left photosensitive region (*G_L_* > *G_R_*), a negative photocurrent (NPC) is generated. As the light spot moves toward the center of the channel (*G_L_* = *G_R_*), the magnitude of the NPC gradually decreases and eventually approaches zero, resulting in a zero photocurrent (ZPC). When the light spot further shifts to the right photosensitive region (*G_L_* < *G_R_*), the photocurrent crosses zero and reverses its polarity, yielding a positive photocurrent (PPC). The corresponding simulation results corroborate this evolution trend (Section S4). The position‐dependent photoresponse can be further abstracted as a vector expressed as [−, 0, +], referred to as the opto‐electronic differential vector (OEDV). The orientation of the OEDV follows the device layout: [−, 0, +] for horizontal alignment and [−, 0, +]ᵀ for vertical alignment.

Figure 1f illustrates the edge extraction mechanism of the CBUP with an OEDV of [−1, 0, 1]. In the dark state, there is no photocurrent signal. When the CBUP is illuminated by light from the right (left) edge of an object, the device produces a PPC (an NPC), whereas uniform illumination leads to a ZPC. Therefore, the CBUP not only enhances signal contrast at object edges but also distinguishes edge orientation based on the polarity of the photocurrent. Furthermore, by tailoring the metallic grating arrangement on the left and right photosensitive regions, the relative magnitudes of the negative photoresponse weight and positive photoresponse weight in the OEDV can be tuned to be unequal (from [−1, 0, 1] to [−1.2, 0, 1]). As a result, the CBUP exhibits a nonzero photocurrent even under uniform illumination, ensuring that information from non‐edge regions of the object is retained, while emphasizing the texture features within the object (Section S5). Therefore, all subsequent measurements were conducted based on the CBUP with an OEDV of [−1.2, 0, 1]. Overall, the CBUP achieves bias‐free, ultra‐broadband operation, and its underlying physical mechanism endows it with inherent sensitivity to intense spatial intensity variations, enabling on‐chip computation‐less edge extraction.

### 2.2 Mechanism Validation

Figure 2a,b show the image of the fabricated 10 × 10 CBUP array. The position‐dependent bi‐directional photoresponse of the CBUP was verified by illuminating different regions with a 1.55 µm laser. The corresponding I‐T curves measured under zero bias at various illumination positions are shown in Figure 2c. When the laser spot is positioned at Area 1 (3), a large number of photocarriers are generated in the left (right) photosensitive region, resulting in an NPC (a PPC). In the case of Area 2, photocarriers collected by both electrodes are nearly equal, and thus the output photocurrent approaches zero. This “negative‐weak‐positive” photoresponse trend is consistent with the simulation results (Section S4) and corresponds to an OEDV of [–, 0, +]. The I‐V curves shown in Figure 2d–f further confirm this trend. In addition, by adjusting the metallic grating configuration of the left/right photosensitive regions, the relative amplitudes of the positive and negative photoresponse can be modulated (Section S7).

**FIGURE 2:**
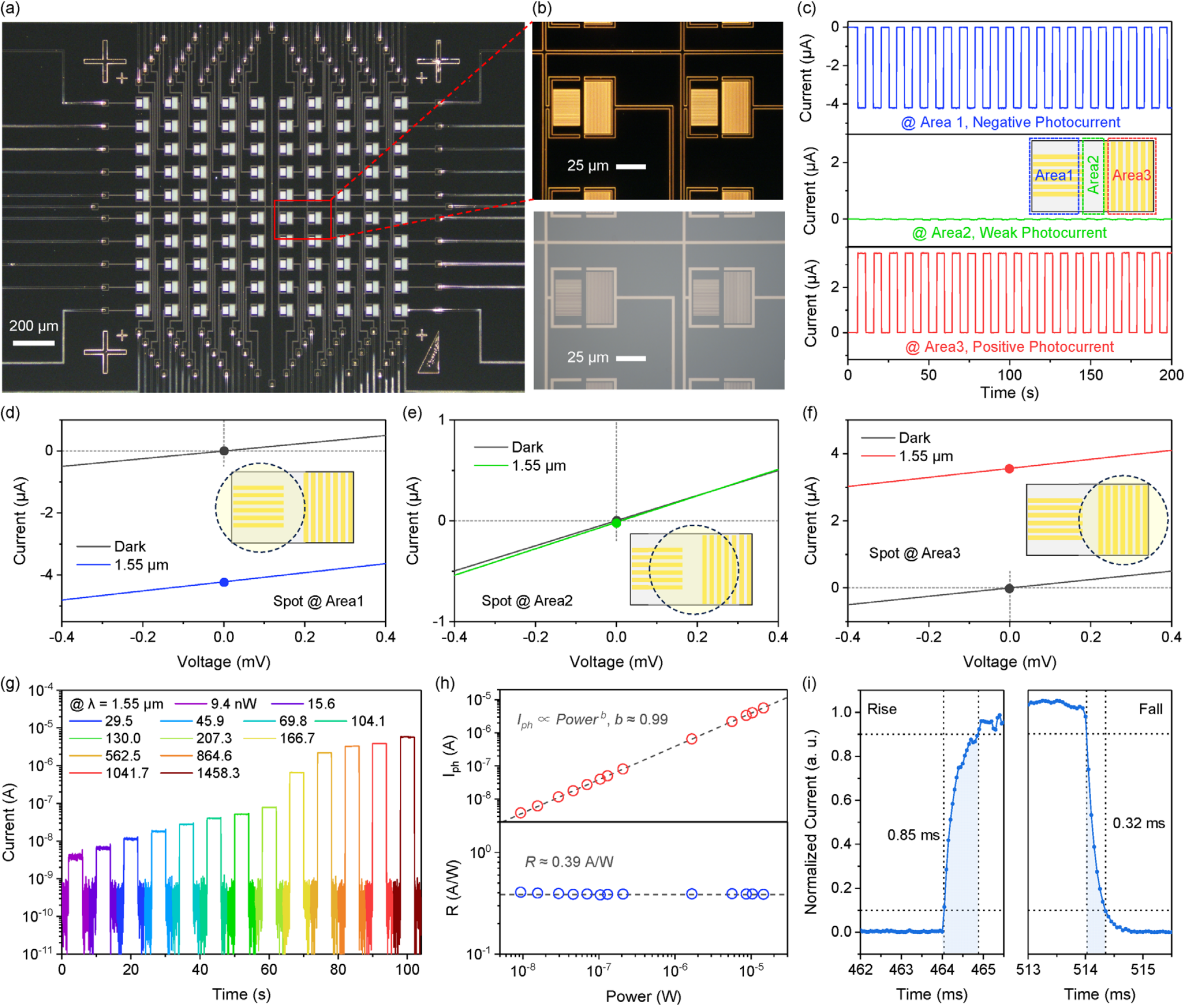
Optoelectronic properties of the CBUP. (a) Dark‐field microscope image of the 10 × 10 CBUP array. (b) A magnified view of the red‐framed region in (a) and the corresponding optical microscope image. (c) Measured I‐T curves of the CBUP under zero bias when a 1.55 µm laser spot is positioned at Area 1, Area 2, and Area 3, respectively. The laser spot diameter was adjusted to ∼50 µm by slightly defocusing the microscope objective. The inset in (c) shows a schematic illustrating the different photosensitive regions. (d–f) Measured I‐V curves of the CBUP under 1.55 µm illumination and in the dark, with the laser spot located at Area 1–3, respectively. (g) Measured I‐T curves of the CBUP at zero bias under 1.55 µm illumination with different optical powers. (h) Top panel: extracted photocurrent as a function of incident 1.55 µm laser power; bottom panel: calculated responsivity as a function of incident power. The minimum detectable optical power of the CBUP is expected to be even lower but is currently limited by the laser source. (i) Measured transient response of the CBUP under zero bias. The center of the 1.55 µm laser spot is positioned in the left photosensitive region in panels (g,h).

Given that the edge extraction capability of the CBUP primarily relates to the photoresponse generated by the target edge signal within the left/right photosensitive regions, positioning the laser spot near either the anode or cathode can be used to evaluate the photodetection performance of the CBUP and its sensitivity to edge signals. For consistency in the photoresponse characterization, except for measurements specifically designed to investigate the position‐dependent bi‐directional photoresponse, the centers of the laser spots at different wavelengths were positioned approximately over the left photosensitive region to generate a negative photoresponse. Figure 2g shows the I‐T curves of the CBUP under 1.55 µm illumination with different powers, where the on‐off ratio reaches as high as ∼3 × 10^4^. In contrast, most photoconductors that rely on external bias suffer from large dark currents, resulting in an on‐off ratio typically below 100 (Section S8). Moreover, over a wide range of optical powers, the photocurrent exhibits a nearly linear dependence on the incident power, with the corresponding responsivity remaining almost constant (Figure 2h). Owing to its high on‐off ratio, the CBUP is expected to achieve a high linear dynamic range of ∼ 89.5 dB. In terms of response speed (Figure 2i), the rise and decay times under zero bias are 0.85 ms and 0.32 ms, respectively, which are sufficient for most imaging applications (∼30 frames per second [23]). Although applying a small external bias can improve the response speed (Section S9), it simultaneously reduces the on‐off ratio, thereby compromising the sensitivity.

### 2.3 Broadband Photoresponse

In addition to exhibiting 1.55 µm near‐infrared (NIR) photoresponse, the CBUP remains responsive to incident light in the infrared range beyond the absorption limit of Ge (λ > 1.8 µm). Figure 3a shows the I‐V curves of the CBUP under 2.7 µm short‐wave infrared (SWIR) illumination, where the maximum short‐circuit current (I_sc_) and open‐circuit voltage (V_oc_) reach 129 nA and 154 µV, respectively (Figure 3b). Unlike the 1.55 µm illumination, which generates photocarriers through the intrinsic absorption of Ge, the photocarriers at 2.7 µm mainly originate from the internal photoemission effect (Section S3.3). Experimental results indicate that the CBUP achieves a maximum responsivity of 35.1 mA/W under 2.7 µm illumination (Section S10). Compared with conventional photoemissive infrared detectors (Section S8), the CBUP not only exhibits a higher responsivity but also operates without cryogenic cooling, which is highly conducive for reducing system size, weight, and power consumption [24, 25].

**FIGURE 3:**
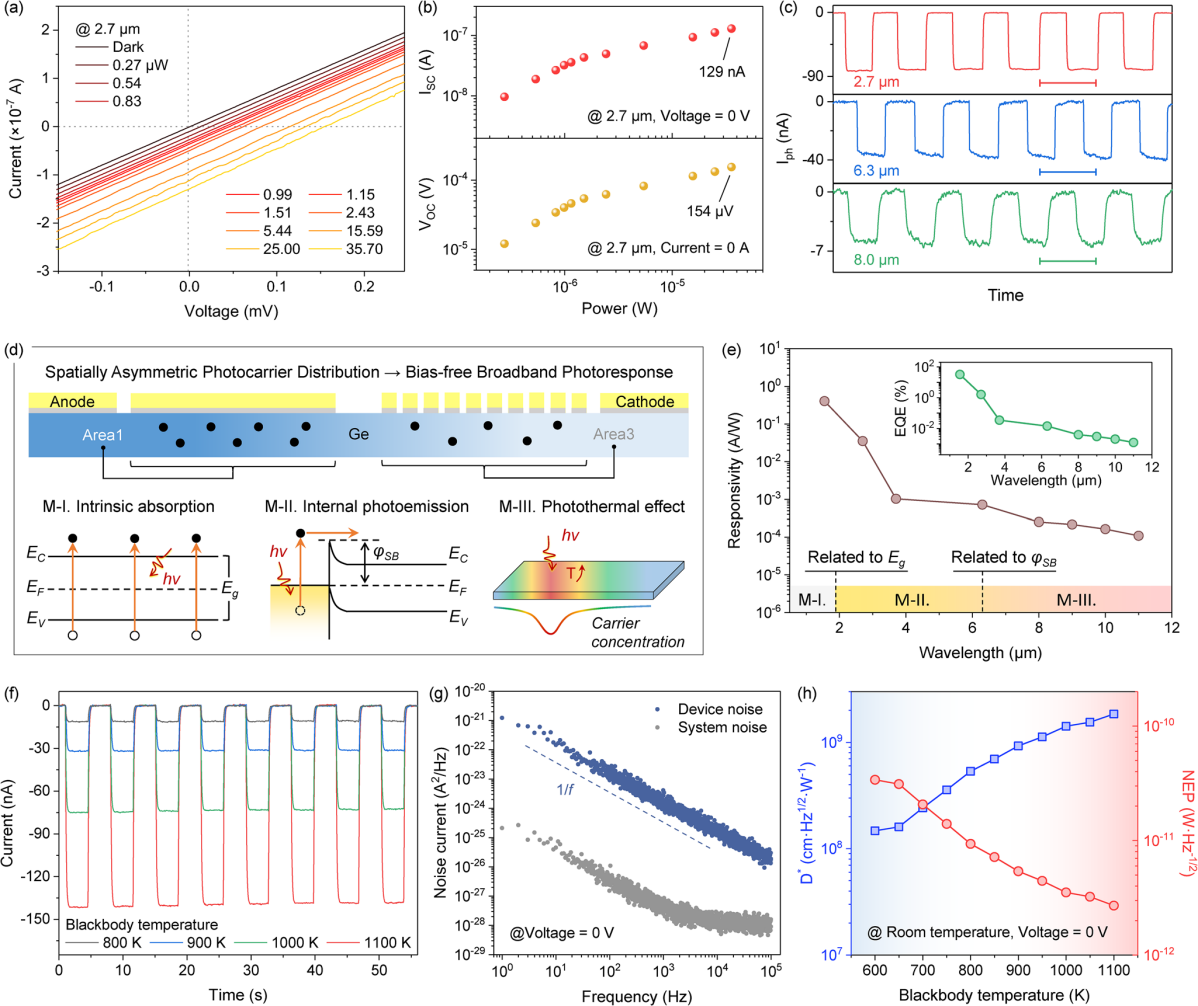
Broadband photoresponse of the CBUP. (a) Measured I‐V curves of the CBUP under 2.7 µm laser illumination with different optical powers. (b) Extracted short‐circuit current and open‐circuit voltage as a function of 2.7 µm laser power. (c) Measured photocurrent of the CBUP under laser illumination with different wavelength (bias voltage is 0 V). Red, blue, and green bar indicates 5.2 s, 10.0 s, and 8.0 s, respectively. (d) Schematic illustration of the three dominant mechanisms for photocarrier generation in the CBUP. (e) Calculated responsivity of the CBUP under illumination at different wavelengths. The dominant photocarrier generation mechanisms in each spectral band are indicated at the bottom. Inset: external quantum efficiency (EQE) as a function of wavelength. (f) Measured zero‐bias I‐T curves of the CBUP under blackbody radiation at different temperatures. (g) Measured noise current of the CBUP measured at 0 V bias. (h) Calculated D^*^ and NEP of the CBUP under blackbody radiation at different temperatures. For broadband photoresponse measurements, the center of the laser spot at different wavelengths is positioned in the left photosensitive region, consistent with the 1.55 µm laser condition in Figure 2g,h. For blackbody response measurements, the blackbody radiation illuminates the entire device.

Furthermore, owing to the internal photoemission effect, the CBUP also responds to mid‐wave infrared (MWIR) light (Figure 3c and Section S11). More importantly, when the MWIR light is incident on different regions of the CBUP, a position‐dependent bi‐directional photoresponse is still observed, indicating that the edge‐detection capability of the CBUP remains effective across a broad spectral range (Section S12). In the long‐wave infrared (LWIR) region, the CBUP continues to operate (Section S13). Since LWIR photons have low energy, the dominant photocarrier excitation mechanism in the CBUP shifts from internal photoemission to the photothermal effect (Section S3.3), which can be verified by the prolonged rise and decay edges of the photocurrent under LWIR illumination [26] (Section S13).

Figure 3d,e summarize the main photocarrier generation mechanisms and photoresponse performance metrics of the CBUP across different spectral regions. The transition between intrinsic absorption and internal photoemission is related to the bandgap (E_g_) of Ge, while the boundary between internal photoemission and photothermal effects is related to the Schottky barrier height (φ_SB_) at the metal‐Ge interface [27]. Although the photocarrier generation mechanism varies with photon energy, the asymmetric metallic grating layout induces spatially nonuniform photocarrier distributions in all cases, which drive measurable photocurrents through the diffusion and bipolar transport of photocarriers. As a result, the CBUP achieves bias‐free and ultra‐broadband photoresponse covering the NIR to LWIR regions.

Given that blackbody radiation is an important equipment for evaluating infrared photodetector performance [28], blackbody sources were employed to further assess the photoresponse capability of the CBUP photoresponse under realistic conditions. Figure 3f and Section S14 show the I‐T and I‐V curves at different blackbody temperatures, where the CBUP exhibits a negative photoresponse. This behavior arises because the blackbody radiation spectrum contains components capable of exciting the intrinsic absorption of Ge, leading to more photocarriers generated in the left photosensitive region and thus producing an NPC. The current noise spectrum displays a typical 1/f noise characteristic (Figure 3g). Based on the measured noise current, the noise‐equivalent power (NEP) and specific detectivity (D^*^) of the CBUP under blackbody radiation are calculated to be 2.71 × 10^−12^ W·Hz^−1/2^ and 1.85 × 10^9^ cm·Hz^1/2^·W^−1^, respectively (Figure 3h), which are comparable to those of emerging high‐performance uncooled low‐dimensional infrared detectors [29, 30, 31, 32, 33, 34].

### 2.4 Demonstration of Edge Detection

In the following, the edge detection capability of the CBUP is demonstrated. By introducing an X‐Y translation stage, scanning imaging can be achieved using a single CBUP (Figure 4a). The test target is a photomask patterned with the letter “G”, where the letter area is transparent, surrounded by a transmissive square frame. First, the CBUP was set to the [−, 0, +]ᵀ state, that is, parallel to the Y‐axis. The photocurrent pattern obtained through scanning is shown in Figure 4b, where the outlines of both the letter “G” and its outer frame can be identified. The scanning pattern obtained by using CBUP reveals edge features and orientations. For example, in Figure 4b, the photocurrent signals at the upper and lower edges are more pronounced than those at the left and right edges, where the upper edge corresponds to a PPC and the lower edge to an NPC. This behavior is consistent with the results obtained using the Y‐Sobel operator (Equation (2), *S*_y_) for 2D convolution. The distinction lies in that Sobel processing requires computational hardware and software, whereas the CBUP achieves computation‐less edge extraction by exploiting its intrinsic physical mechanism. Note that, when a bias is applied, the CBUP behaves as a conventional photoconductor, and the scanning photocurrent pattern only reflects the light intensity distribution (Section S15).

(2) $$S_{y}=\left[\begin{array}{ccc} -1 & -2 & -1\\ 0 & 0 & 0\\ 1 & 2 & 1 \end{array}\right],\;S_{x}=\left[\begin{array}{ccc} -1 & \;0 & \;1\\ -2 & \;0 & \;2\\ -1 & \;0 & \;1 \end{array}\right]$$

**FIGURE 4:**
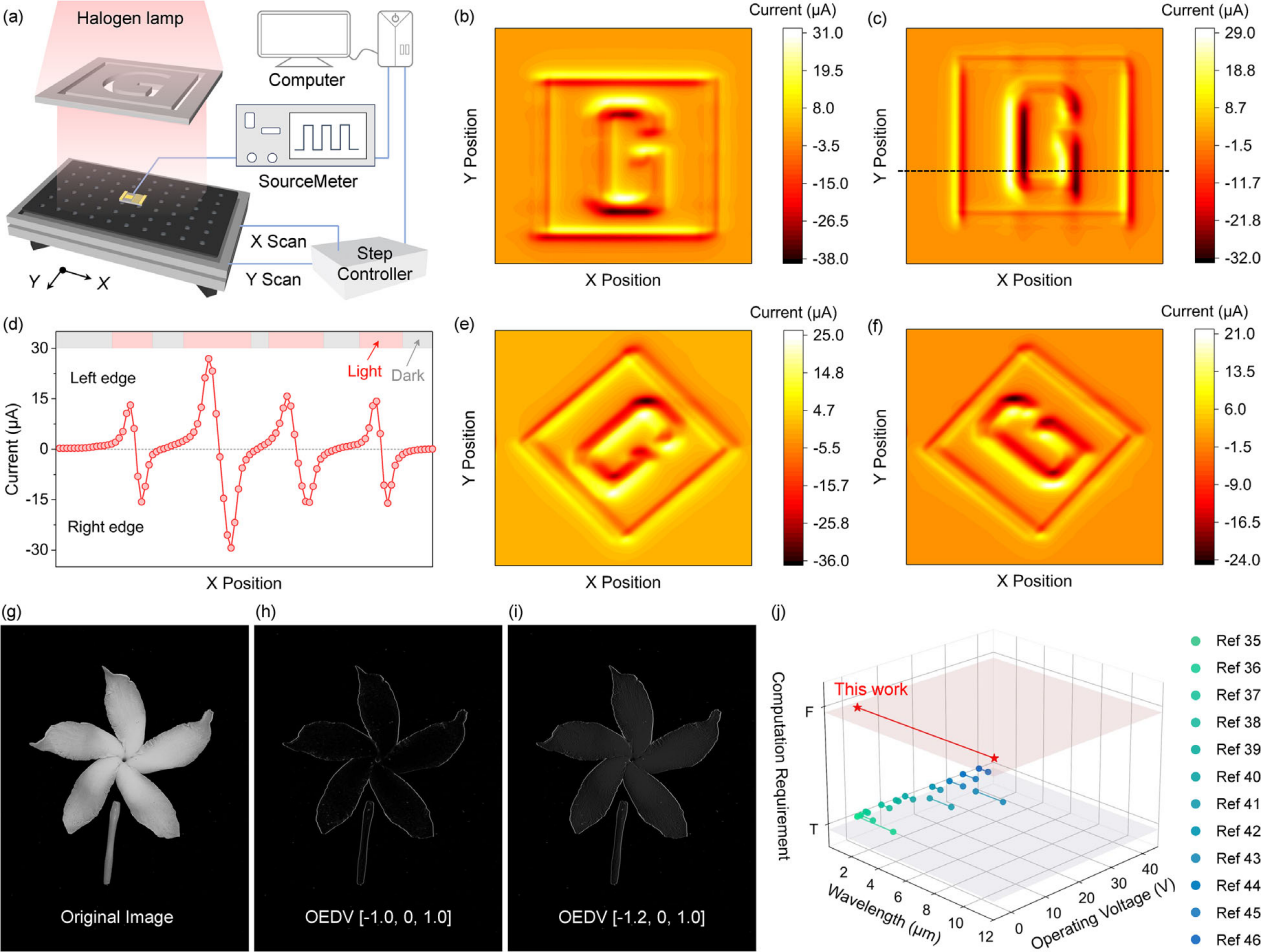
Demonstration of edge extraction using the CBUP. (a) Schematic illustration of the scanning setup based on a single CBUP. (b,c) Experimentally measured scanning photocurrent pattern obtained with the CBUP in [−, 0, +]^T^ and [−, 0, +] form, respectively. (d) Line profile extracted from (c). Due to scattering from the halogen lamp, the photocurrent signal around the edges of the target decays slowly. (e,f) Experimentally measured scanning photocurrent pattern acquired with the CBUP in [+, 0, −]^T^ form, with the mask rotated by approximately −45° and +45°, respectively. (g–i) Edge‐extraction simulation results based on different OEDVs. From left to right: original image, and edge images obtained with OEDVs of [−1, 0, +1] and [−1.2, 0, +1], respectively. (j) Summary of emerging photodetectors for on‐chip edge extraction [35, 36, 37, 38, 39, 40, 41, 42, 43, 44, 45, 46].

Next, the CBUP was rotated to the [−, 0, +] state, that is, parallel to the X‐axis. The corresponding scanning results are shown in Figure 4c. In this configuration, the left and right edge signals become stronger, while the upper and lower edges are suppressed, consistent with the numerical results of the X‐Sobel operator (Equation (2), *S*_x_). Figure 4d presents the data extracted along the dashed line in Figure 4c, where the PPC and NPC correspond to the left and right edges, respectively, indicating the position and orientation of the detected edges (Section S16). Furthermore, when the CBUP was set to the [+, 0, −]ᵀ state (parallel to the Y‐axis) and the photomask was rotated by +45° and −45°, the scanning results shown in Figure 4e,f can be obtained. Compared with Figure 4a, due to a 180° rotation of the CBUP, the upper edge now corresponds to an NPC and the lower edge to a PPC. Meanwhile, because the CBUP and photomask edges were misaligned by a certain angle, all edges of the letter pattern and its outer frame were effectively extracted.

Since the position‐dependent bi‐directional photoresponse of the CBUP can be abstracted as an OEDV of [−, 0, +], and the weights of positive and negative photoresponses can be tuned by adjusting the metallic grating layout (Section S7), various OEDV can be constructed to simulate different types of CBUPs. To demonstrate simulation‐based edge extraction, two OEDVs were constructed with weight parameters of [−1, 0, 1] and [−1.2, 0, 1], which approximately corresponds to devices with left/right grating ratios of 3:3 and 2:3, respectively. Notably, the photocurrent pattern of a 10 × 10 CBUP array matches the simulated results, confirming the feasibility of using OEDVs to emulate real devices (Section S17). Based on the above OEDVs, the edge information of the target image (Figure 4g) can be obtained by simulation. As shown in Figure 4h,i, the simulated results were processed by taking their absolute values, followed by normalization and conversion to the 0–255 grayscale range for visualization. When the positive and negative photoresponse weights are close, the images exhibit finer edge details; as the weight difference increases, the overall texture becomes more prominent. These results indicate that by tailoring the grating layout to adjust the photoresponse weighting, different edge extraction effects can be flexibly achieved according to specific requirements.

## 3 Conclusion

In conclusion, this work demonstrates an edge‐sensitive photoconductor based on a metallic‐grating‐decorated Ge structure. Compared with other emerging reconfigurable photodetectors for on‐chip edge extraction (Figure 4j, Section S18), the proposed device, featuring bias‐free and computation‐less operation, offers a remarkably simple architecture and superior energy efficiency. In addition, it exhibits an ultra‐broadband photoresponse ranging from the NIR to LWIR at room temperature, and shows commendable sensitivity to blackbody radiation. By directly performing spatial differentiation along the OEDV direction at the device‐physics level and outputting signals related to edge intensity and orientation, the CBUP can reduce the need for frequent raw‐data transmission, storage, and numerical convolution in conventional edge‐extraction workflows. More importantly, this device enables direct perception of edge features at the single‐pixel level, whereas conventional numerical‐computation‐based edge extraction requires integrating signals from multiple neighboring pixels.

It is noteworthy that such position‐dependent bidirectional photoresponse is expected to be widely present in various photoconductor systems. Therefore, the proposed design concept is not limited to Ge and can be extended to other semiconductor platforms, including silicon, thereby providing a potential pathway toward future CMOS‐compatible integration. Although numerical edge extraction remains indispensable in current computer vision systems, this work reveals the potential of physics‐driven edge extraction at the device level, offering a new perspective and pathway toward more efficient and compact visual perception systems.

## 4 Experimental Section

### 4.1 Device Fabrication

The fabrication of the CBUPs started with an intrinsic Ge wafer (100 Ω·cm). Metallic gratings and alignment markers were fabricated with electron‐beam lithography (EBL; JBX‐6300FS, Jeol), electron‐beam evaporation of 3/50‐nm Ti/Au (AJA International), and lift‐off in hot acetone at 65°C for 2 h. EBL process comprised spin‐coating of poly(methyl methacrylate) (PMMA) 495 A5 at 4,000 r.p.m., baking at 180°C for 1 min, with an exposure dose of 1300 µC·cm^−2^; development in methyl isobutyl ketone: isopropyl alcohol (MIBK:IPA) = 1:3 for 10 s and a rinse in IPA for 30 s. Afterwards, contact electrodes were patterned onto the Ge by using a laser writer (LW405B, MICROTECH), followed by electron‐beam evaporation (3/50‐nm Ti/Au). Finally, the samples were soaked into hot Remover PG at 65°C for 2 h to perform lift‐off and obtain the CBUPs.

### 4.2 Device characterization

The electric characteristics (I‐V, I‐T, noise) of the CBUPs was measured by a semiconductor device analyser (FS‐Pro, PRIMARIUS). Several light sources, including 1.55 µm laser, 2.7 µm laser (Thorlabs), MWIR quantum cascade laser, LWIR quantum cascade laser (Daylight Solution, MIRCat), and blackbody (SR‐200N) were used in this work. Laser power at 1.55 µm was calibrated using a commercial Ge photovoltaic detector (GMTL 10HS TO‐9). The optical powers at the other wavelengths were measured using a commercial broadband optical power meter (Thorlabs S405C). The scanning photocurrent mapping was acquired using a displacement stage (Marzhauser Wetzlar), a halogen lamp (U‐LH100L‐3, OLYMPUS) and a source meter (2614B, Keithley). The periodic switching of the light source was realized by exploiting a signal generator (Rigol DG1022U) or an opto‐electronic shutter (Daheng Optics). All measurements were performed at room temperature and under ambient conditions.

### 4.3 Simulation

The optical features of the designed metallic gratings were simulated with the finite‐difference time‐domain method (Section S1). The carrier distribution, current vector, and position‐dependent photoresponse of the CBUP were obtained using technology computer‐aided design (TCAD) software simulation. The physical models adopted in the TCAD simulations are described in detail in SI Section S4. The binary image and grayscale flow image used in the experiment were generated on the computer and photographed by author, respectively. All edge extraction simulations were implemented by Python code.

## Author Contributions

J.F., X.W., and C.‐W.Q. conceived the project. J.F. carried out the theoretical analysis and numerical simulations. J.F. fabricated the samples. J.F. carried out and contributed to the device characterization with the help of H.J., F.W., and Y.P. J.F., X. L., Q.W., W.G., X.W., and C.‐W.Q. discussed and analyzed the numerical and experimental results. All authors discussed and contributed to the manuscript. C.‐W.Q. oversaw the whole project.

## Conflicts of Interest

The authors declare no conflicts of interest.

## Supporting information




**Supporting File**: adma74925‐sup‐0001‐SuppMat.docx.

## Data Availability

The data that support the findings of this study are available from the corresponding author upon reasonable request.
