# Supplementary material for: Computation‐Less, Bias‐Free, and Ultra‐Broadband Photodetector for On‐Chip Edge Extraction
Source: Adv Mater. 2026 Sep 11;38(61):e74925. doi: 10.1002/adma.74925 (PMC13726709; doi:10.1002/adma.74925)
Supplement: Supplementary file 1 [file ADMA-38-e74925-s001.docx]

*Supporting Information*

Computation-less, Bias-free, and Ultra-broadband Photodetector for On-chip Edge Extraction

*Jintao Fu*^1^, *Hao Jiang*^2^, *Xingsi Liu*^1^, *Fakun Wang*^2^, *Yang Peng*^3^, *Qi Jie Wang*^2^, *Weibo Gao*^2^, *Xingzhan Wei*^3, #^, *Cheng-Wei Qiu*^1, #^

^1^Department of Electrical and Computer Engineering, National University of Singapore, Singapore, Singapore.

^2^School of Electrical and Electronic Engineering, Nanyang Technological University, Singapore, Singapore

^3^Chongqing Institute of Green and Intelligent Technology, Chinese Academy of Sciences, Chongqing, People’s Republic of China.

^#)^ Corresponding author. Emails: [weixingzhan@cigit.ac.cn](mailto:weixingzhan@cigit.ac.cn), [chengwei.qiu@nus.edu.sg](mailto:chengwei.qiu@nus.edu.sg).

**Table of Contents**

[▪	Section 1. Subwavelength Metallic Grating Structure	3](#_Toc238105265)

[▪	Section 2. Device Design Inspired by Mathematical Principles	5](#_Toc238105266)

[▪	Section 3. Operation Mechanism of the CBUP	11](#_Toc238105267)

[▪	Section 4. Position-dependent Bi-directional Photoresponse	25](#_Toc238105268)

[▪	Section 5. Symmetry-enabled Bias-free Photodetection	27](#_Toc238105269)

[▪	Section 6. Edge Extraction Based on CBUPs with Different OEDVs	29](#_Toc238105270)

[▪	Section 7. CBUPs with Different Grating Coverage Ratios	30](#_Toc238105271)

[▪	Section 8. Performance Summary	35](#_Toc238105272)

[▪	Section 9. NIR Photoresponse of the CBUP	37](#_Toc238105273)

[▪	Section 10. SWIR Photoresponse of the CBUP	39](#_Toc238105274)

[▪	Section 11. MWIR Photoresponse of the CBUP	40](#_Toc238105275)

[▪	Section 12. Position-Dependent WMIR Bi-directional Photoresponse	42](#_Toc238105276)

[▪	Section 13. LWIR Photoresponse of the CBUP	44](#_Toc238105277)

[▪	Section 14. Blackbody Radiation Response Measurement	48](#_Toc238105278)

[▪	Section 15. Scanning photocurrent pattern under applied bias voltage	51](#_Toc238105279)

[▪	Section 16. Edge Extraction Performance Evaluation	52](#_Toc238105280)

[▪	Section 17. Photoresponse of the CBUP Array	57](#_Toc238105281)

[▪	Section 18. Photodetectors for On-chip Edge Extraction	62](#_Toc238105282)

- **Section 1. Subwavelength Metallic Grating Structure**

The subwavelength metallic grating in the photosensitive region of the CBUP was designed based on the peak spectral response wavelength of the Ge channel material (approximately 1550 nm), aiming to achieve strong coupling between the grating and the incident light at this wavelength. To this end, finite element simulations were performed for the metallic grating-Ge structure. By comparing the light absorption efficiencies under TM and TE polarized light, the metallic grating geometry for fabrication was determined. Here, TM light refers to incident light with an electric field polarized perpendicular to the grating lines, while TE light refers to polarization parallel to the grating lines. In the simulations, perfectly matched layers (PML, N = 16) were applied as boundary conditions above and below the grating array. The simulated ratio of TM to TE absorption is shown in Figure S1. The results indicate that when the grating period is 1.4 μm and the metallic grating width is approximately 0.8 μm, the absorption ratio between the two orthogonal polarizations reaches its optimized state. Therefore, these parameters were selected for the grating fabrication.

In, addition, compared with bare Ge, the introduction of the Ti/Au grating can significantly enhance the absorption of the overall structure in a broad spectral range from near-infrared to long-wave infrared (Figure S2). Moreover, Ti interlayer improves the adhesion of Au on the Ge substrate, ensuring reproducible metal grating fabrication.

**Figure S1. Optical simulation of the metallic grating.** (a-c) Ratios of optical absorption between TM and TE polarized light at different wavelengths and metal grating lengths, with grating periods of 1.2 μm, 1.3 μm, and 1.4 μm, respectively.

**Figure S2. Simulated optical absorption of the Bare Ge and Au/Ti-Ge structures.** (a, b) Absorption spectra under broadband TM- and TE-polarized incident light, respectively. (c, d) Corresponding increase multiplier (IM) distributions, where IM is calculated as the ratio of the absolute absorption of the Au/Ti-Ge structure to that of the Bare Ge structure. In the simulations, the metallic grating period is 1.4 μm, the metallic stripe width is 0.8 μm, the Ti thickness is 3 nm, and the Au thickness is 50 nm. The T-window is located 0.3 μm below the Ge surface. The R-window is located 1.5 μm above the Ge surface, respectively, while the light source is positioned 1 μm above the Ge surface. The optical absorption (A) is calculated as A = 1 - R - T. According to the simulation results, the Bare Ge structure exhibits negligible absorption over a broad wavelength range, whereas the introduction of the Au/Ti grating significantly enhances the absorption across the broad spectral band. The curves in (a) are reused in Figure 1d.

- **Section 2. Device Design Inspired by Mathematical Principles**

In digital image processing, an edge is commonly defined as the location where the first-order derivative of the image intensity function reaches an extremum or where the second-order derivative crosses zero. As illustrated in Figure S3, when an edge exists in a scene, the image intensity exhibits a sharp variation. Consequently, the first-order derivative of intensity with respect to position reaches a maximum or minimum (depending on the edge direction), while the second-order derivative passes through zero. In contrast, for regions with uniform illumination or complete darkness, where the intensity remains constant (either 0 or 1), both the first- and second-order derivatives of the intensity are zero everywhere.

**Figure S3. Different light intensity distribution functions and their derivatives.** (a-c) Calculated light intensity as a function of position under uniform illumination, edge, and dark-state conditions, respectively. (d-f) Calculated first derivatives of the light intensity functions shown in (a-c). (g-i) Calculated second derivatives of the light intensity functions shown in (a-c).

Assuming a two-dimensional image function *f* (*x*, *y*), where (*x*, *y*) denotes the pixel coordinates, the gradient of the image can be defined as:

$$\nabla f=\left[\begin{matrix} \frac{\partial f}{\partial x} \\ \frac{\partial f}{\partial y} \end{matrix}\right]=\left[\begin{matrix} f_{x} \\ f_{y} \end{matrix}\right]$$

The gradient magnitude, representing the edge strength, is given by $\left|\nabla f\right|=\sqrt{f_{x}^{2}+f_{y}^{2}}$, while the gradient direction, indicating the edge normal, is expressed as $\theta=\tan^{-1}\left(f_{y}/f_{x}\right)$.

For digital images, the derivatives can be discretized using finite differences, typically approximated as:

$$\frac{\partial f}{\partial x}=f\left(x+1,y\right)-f\left(x,y\right),\text{ }\frac{\partial f}{\partial y}=f\left(x,y+1\right)-f\left(x,y\right)$$

It is evident that performing finite-difference operations on sampled signals is an effective method for edge extraction. The simplest form is the two-point difference operator [-1, 1], while an optimized version, the three-point central difference operator [-1, 0, 1], offers higher edge localization accuracy and better noise resistance. The frequency-domain behavior of the three-point central difference operator can be analyzed using the discrete-time Fourier transform (DTFT). For a discrete sequence *h*[*n*], the DTFT is defined as:

$$H\left(\omega\right)=\sum_{n=-\infty}^{+\infty} h\left[n\right]\times e^{-j\omega n}$$

where *ω* is the digital angular frequency ranging from -π to π, *j* is the imaginary unit, and *e*^−^*^jωn^* represents a complex phase shift. Substituting *h*[-1] = -1, *h*[0] = 0, and *h*[1] = 1 into the DTFT formula and combining it with Euler's formula, following equation can be obtained,

$$H\left(\omega\right)=-e^{j\omega}+e^{-j\omega}=-2j\sin\left(\omega\right)$$

The magnitude response |H(ω)| = 2|sin(ω)| indicates that this operator suppresses the DC component (*ω* = 0, corresponding to regions with constant light intensity), allows the mid-frequency components (*ω* ≈ π/2) corresponding to edge information to pass, and attenuates high-frequency components (*ω* → π), which typically correspond to random noise such as shot or speckle noise. Hence, the [-1, 0, 1] operator functions as a band-pass filter that suppresses low-frequency background and high-frequency noise, while preserving mid-frequency edge information.

Although the digital angular frequency *ω* is mathematically defined over [-π, π], for real-valued signals, the spectrum is symmetric about zero, and thus analysis is typically restricted to the interval [0, π]. Here, *ω* = π corresponds to the Nyquist frequency (*f_N_* = *f_s_* / 2), the highest representable frequency in the sampling system. When *ω* approaches π, the signal nears the sampling limit, and higher frequencies may be distorted due to aliasing.

Inspired by above mathematical principles, a physical implementation for the three-point central difference operator using a single photodetector to achieve on-chip edge extraction is proposed in the work. Through position-dependent bi-directional photoresponse, “positive”, “zero”, and “negative” photoresponse weights are realized. Since both photocurrents share a common conduction channel, they interact and combine, effectively performing a physical convolution. Therefore, the designed device reproduces the behavior of a [-, 0, +] operator, which is referred to as the opto-electronic differential vector (OEDV).

OEDV is introduced as a discrete representation of the position-dependent bi-directional photoresponse of the CBUP. For an ideal OEDV such as [-1, 0, +1], its mathematical form corresponds to a discrete first-order central-difference operator along a specific spatial direction and therefore provides an approximate measure of the local optical-intensity gradient. In practical devices, however, the positive and negative response amplitudes may not be identical, resulting in asymmetric OEDVs such as [-a, 0, +b], |a| ≠ |b|. Such an OEDV still represents a weighted spatial-difference operation but is not strictly identical to the standard first-order central-difference operator.

It should also be emphasized that the OEDV is a discrete equivalent representation of the device response rather than the complete continuous physical response itself. In other words, the OEDV captures the dominant spatial-differentiation behaviour of the CBUP. The experimentally measured photocurrent is determined by the spatial distribution of the incident optical intensity, the position-dependent response function of the device, and carrier transport over the finite device area. The OEDV discretizes this continuous spatial response into a finite set of weights for numerical modelling and image simulation.

Analogous to linear algebraic vectors, the OEDV exists in two forms, [-, 0, +] and [-, 0, +]^T^, corresponding to edge extraction in horizontal and vertical directions, respectively. When the device is horizontally oriented, it enhances horizontal edge extraction, and when vertically placed, it enhances vertical edge extraction. Moreover, in practice, the device orientation is not limited to 0° and 90°; it can be rotated to arbitrary angles for extracting edges along any direction.

Furthermore, by adjusting the spatial configuration of the subwavelength metallic gratings, the magnitudes of the positive and negative photoresponse weights can be asymmetrically tuned, for example, from [-1, 0, 1] to [-1.2, 0, 1]. Substituting *h*[-1] = -1.2, *h*[0] = 0, and *h*[1] = 1 into the DTFT formula gives:

$$H\left(\omega\right)=-1.2e^{j\omega}+e^{-j\omega}=e^{-j\omega}\left(1-1.2e^{j2\omega}\right)$$

and the corresponding magnitude:

$$\left|H\left(\omega\right)\right|^{2}=\left|1-1.2e^{j2\omega}\right|^{2}=1+1.2^{2}-2\cdot1\cdot1.2\cos\left(2\omega\right)=2.44-2.4\cos\left(2\omega\right)$$

$$\left|H\left(\omega\right)\right|=\sqrt{2.44-2.4\cos\left(2\omega\right)}$$

This asymmetric operator [-1.2, 0, 1] still exhibits its strongest response near mid-frequencies, but allows a certain portion of low-frequency components to pass, thus preserving some non-edge information instead of losing it completely.

Figure S4 illustrates the amplitude and phase distributions of the OEDV [-1.2, 0, 1] when oriented at 0°, 45°, 90°, and 135°. For clarity, these orientations can be represented in matrix form as shown below.

$$\left[\begin{matrix} 0 & 0 & 0 \\ -1.2 & 0 & 1 \\ 0 & 0 & 0 \end{matrix}\right],\text{ }\left[\begin{matrix} 0 & -1.2 & 0 \\ 0 & 0 & 0 \\ 0 & 1 & 0 \end{matrix}\right],\text{ }\left[\begin{matrix} -1.2 & 0 & 0 \\ 0 & 0 & 0 \\ 0 & 0 & 1 \end{matrix}\right],\text{ }\left[\begin{matrix} 0 & 0 & -1.2 \\ 0 & 0 & 0 \\ 1 & 0 & 0 \end{matrix}\right]$$

From the amplitude maps, it can be observed, consistent with the previous frequency-domain analysis, that the strongest responses occur in the mid-frequency region, highlighting the sensitivity of the OEDV to edge features. These high-response regions rotate correspondingly with the vector orientation, indicating that edge extraction along different directions can be selectively enhanced by simply rotating the device. Furthermore, in these strong-response regions, the phase values are approximately ±π/2, indicating the direction of intensity transitions, either from bright to dark or from dark to bright.

**Figure S4. Frequency-domain analysis of the OEDV [-1.2, 0, 1].** (a-d) Calculated amplitude distributions of the OEDV oriented at 0°, 45°, 90°, and 135°, respectively. (e-h) Corresponding calculated phase distributions of (a-d).

As discussed above, edge information along different orientations can be extracted by varying the relative angle between the CBUP and the target edge. This observation indicates that the edge response of the CBUP depends on the relative edge angle. Considering that the position-dependent bi-directional photoresponse of the CBUP can be equivalently described using an OEDV, the *x*-direction OEDV [-1, 0, +1] was selected as a representative example to theoretically analyse the angular dependence of the edge response.

In the model, the edge angle *θ* is defined as the angle between the edge normal and the +*x* direction, with the edge normal pointing from the dark region toward the bright region (Figure S5a). A two-dimensional ideal step edge can be expressed as follows:

$$I\left(x,y;\theta\right)=I_{d}+\Delta I\cdot H\left(x\cos\theta+y\sin\theta-d\right)$$

where *I_d_* is the grayscale intensity of the dark region, ∆*I* = *I_b_* - *I_d_* is the grayscale intensity difference between the bright and dark regions, *H*(·) is the Heaviside step function, and *d* describes the position of the edge relative to the image centre. Because an ideal step edge is not differentiable at the boundary, it can be represented using the following smoothed form:

$$I\left(x,y;\theta\right)=I_{d}+\frac{\Delta I}{2}\cdot\left[1+\tanh\left(\frac{s}{\sigma}\right)\right]$$

where (*s* = *x*cos*θ* + *y*sin*θ* - *d*), and *σ* represents the edge-transition width.

Because the OEDV [-1, 0, +1] is equivalent to a first-order spatial difference along the *x* direction, its output can be described, under the continuous approximation, by the partial derivative of the image intensity with respect to *x*:

$$R_{x}\left(\theta\right)=\partial I/\partial x=\frac{\Delta I}{2\sigma}\cdot\text{sech}^{2}\left(\frac{s}{\sigma}\right)\cdot\cos\theta$$

This expression consists of two components. The first component, (△*I* / 2*σ*)·[sech(*s*/*σ*)]^2^, describes the local magnitude of the edge gradient. It reaches its maximum at the edge centre, where *s* = 0, and gradually approaches zero as the position moves away from the edge. The second component, cos*θ*, represents the projection of the edge gradient onto the *x* direction. Therefore, near the edge centre, the response can be simplified as,

$$R_{x}\left(\theta\right)=C\Delta I\cdot\cos\theta$$

where *C* is related to the edge-transition width.

According to this expression, the signed edge response of the CBUP approximately follows a cos*θ* dependence (Figure S5b). At *θ* = 0°, the response reaches its maximum positive value. As *θ* increases from 0° to 90°, the positive response gradually decreases to zero. The response then becomes negative and reaches its maximum negative value at (*θ* = 180°). It decreases in magnitude to zero again at (*θ* = 270°) and finally returns to its maximum positive value at (*θ* = 360°). When only the photocurrent contrast or response magnitude is considered, the angular dependence approximately follows (|cos*θ*|), whereas the positive and negative photocurrent polarities can be used to distinguish oppositely oriented edges.

**Figure S5. Angel-dependent edge response.** (a) Schematic diagram of the edge at angle *θ*. (b) Theoretically calculated angle-dependent edge response when the OEDV [-1, 0, +1] is along the *x* direction.

Although the above analysis fixes the OEDV along the *x* direction and rotates the image edge, rotating the CBUP or its OEDV orientation in experiments is equivalent to changing the relative angle between the device and the edge. Therefore, adjusting the orientation of the CBUP enables the selective extraction of edge features along different directions. To simultaneously acquire two orthogonal gradient components from a two-dimensional scene in a single acquisition, two adjacent devices with mutually orthogonal OEDVs can be integrated as a macropixel. The two devices separately output edge responses along the two orthogonal directions, after which the two-dimensional edge magnitude can be obtained through simple absolute-value, summation, or gradient-magnitude operations. This array design enables multidirectional edge sensing in a single acquisition while retaining device-level spatial differentiation.

- **Section 3. Operation Mechanism of the CBUP**

**3.1 Mechanism of the Bias-Free Photoresponse**

The generation of a stable photoresponse under bias-free conditions in the CBUP can be qualitatively described by the following stages.

**Stage I.** In the absence of an external bias, the Ge channel experiences a zero internal electric field (*E* ≈ 0). Under the dark condition, the electron and hole concentrations are solely determined by the doping of Ge and remain in thermal equilibrium.

**Stage II.** Upon illumination, the asymmetric grating configurations in the left and right photosensitive regions lead to an imbalance in the number of photocarriers. This can be described by different photocarrier generation rates, *G_L_* and *G_R_*, in the two regions, resulting in a spatially non-uniform carrier distribution along the channel. For example, when *G_L_* > *G_R_*, both electron and hole concentrations are higher in the left region than in the right one. In the absence of an external electric field, carrier transport is initially dominated by diffusion, with both electrons and holes diffusing from regions of higher concentration toward regions of lower concentration.

**Stage III.** However, due to the difference between electron and hole mobilities (*μ_n_* ≠ *μ_p_*), the diffusion process becomes asymmetric. In Ge, where *μ_n_* > *μ_p_*, electrons diffuse faster and over longer distances than holes. As a result, under steady illumination, electrons accumulate preferentially in the low-*G* region, while excess holes remain in the high-*G* region. This spatial separation of charges establishes positively and negatively charged regions across the left and right photosensitive areas. The resulting space charge induces a lateral electric field within the channel via Poisson’s equation. Owing to its similarity to the conventional Photo-Dember effect, this induced lateral field is referred to here as a static Photo-Dember field.

Under open-circuit conditions, the total current must vanish (*J* = 0). Consequently, the static Photo-Dember field self-consistently adjusts to counterbalance the diffusion current driven by carrier concentration gradients. The integral of this field along the channel corresponds to the open-circuit photovoltage, *V_OC_*.

Under short-circuit conditions, the potentials at the two electrodes are equalized by the metallic connection. In this case, the system must re-establish equilibrium between diffusion-driven transport and the boundary constraint imposed by the electrode potentials. On the one hand, the static Photo-Dember field remains determined by the asymmetric generation rates and mobility difference. On the other hand, the finite conductivity of the channel allows a steady current to flow, producing a voltage drop along the channel that compensates the terminal potential difference associated with the static Photo-Dember field. This balance ultimately manifests as a steady direct current, namely the short-circuit photocurrent *J_SC_*.

Moreover, because the Dember field and diffusion current depend on the spatial distribution of the photocarrier generation rate, changing the illumination position reverses the carrier-concentration gradient and the direction of the short-circuit current, thereby producing a position-dependent bi-directional photoresponse. The position-dependent bi-directional photoresponse forms the physical basis for device-level spatial differentiation and edge extraction in the CBUP. Since the above process occurs laterally within the Ge thin film and produces a steady-state output under continuous illumination, it is described here as the transverse static Photo-Dember effect.

Although the pn-junction photovoltaic effect, the conventional Photo-Dember effect, and the transverse static Photo-Dember effect discussed in this work can all generate photoinduced electrical signals without an externally applied bias, their physical origins and operating states differ. In the pn-junction photovoltaic effect, carrier diffusion between the p-type and n-type regions forms a depletion region and a junction built-in field under thermal-equilibrium conditions. Photogenerated electron-hole pairs are then separated by this pre-existing electric field, producing a photoresponse output.

By contrast, the Photo-Dember effect does not rely on a pre-formed pn junction but originates from differences between the electron and hole diffusion coefficients or mobilities under nonuniform optical excitation. Their unequal diffusion rates cause spatial separation of the photogenerated carriers, thereby producing a Dember potential and electric field. Under ultrafast pulsed excitation, this process commonly manifests as a rapidly varying transient photocurrent or dipole moment and can generate terahertz radiation. Under continuous nonuniform illumination, however, it can also produce a photoinduced potential and steady-state carrier transport.

**3.2 Model for the Bias-Free Photoresponse**

In this subsection, a model for the bias-free steady-state photoresponse is derived. This model aims to explain both the simulation results and the experimental observations, specifically addressing the following two questions:

(1) Why does a steady photocurrent emerge under zero external bias (i.e., bias-free photoresponse)?

(2) Why does the polarity of the photocurrent reverse when the photocarrier generation rates in the left and right photosensitive regions are interchanged (i.e., position-dependent bi-directional photoresponse)?

A one-dimensional semiconductor channel of length *L* along the *x*-direction is considered, in which the left and right photosensitive regions each occupy half of the channel. The contacts at both ends are assumed to be symmetric Ohmic contacts, consistent with the experimental observations. The semiconductor is intrinsic Ge, such that under dark conditions the equilibrium carrier concentrations satisfy *n_0_* = *p_0_* = *n_i_*. Optical excitation is treated within the low-to-moderate injection regime, where the excess carrier densities satisfy ∆*n* = ∆*p* << *n_i_*.

Due to the asymmetric metallic grating configurations in the left and right photosensitive regions, the numbers of photocarriers differ spatially. This asymmetry can be equivalently described by different photocarrier generation rates in the two regions. Specifically, the generation rates are approximated as piecewise constants:

$$\begin{matrix} G\left(x\right)=G_{L},\text{ }0<x<L/2 \\ G\left(x\right)=G_{R},\text{ }L/2<x<L \end{matrix}$$

where *G_L_* and *G_R_* denote the photocarrier generation rates in the left and right halves of the channel, respectively.

**Task 1: Introduction of Standard Relations**

Based on the drift-diffusion formalism, the electron current density (*J_n_*) and hole current density (*J_p_*) can be written as

where *μ_n_* and *μ_p_* are the electron and hole mobilities; *n* and *p* are the electron and hole concentrations; *E* is the electric field; *q* is the elementary charge; and *D_n_* and *D_p_* are the electron and hole diffusion coefficients.

For an intrinsic semiconductor, the electron and hole concentrations are approximately equal, i.e., n(x) ≈ p(x). Under this condition, the total current density *J* can be expressed as

$$\begin{matrix} J=J_{n}+J_{p} \\ J=qn\left(\mu_{n}+\mu_{p}\right)E+q\left(D_{n}-D_{p}\right)\frac{dn}{dx} \end{matrix}$$

The diffusion coefficients *D_n,p_* and mobilities *μ_n,p_* are related through the Einstein relation, *D* = *μk_B_T*/*q*, where *k_B_* is the Boltzmann constant and *T* is the temperature.

**Task 2: Expression for the Open-Circuit Voltage V_OC_**

Under open-circuit conditions, the total current density vanishes, i.e., *J* = 0. Substituting this condition into the expression for the total current density yields,

$$0=qn\left(\mu_{n}+\mu_{p}\right)E+q\left(D_{n}-D_{p}\right)\frac{dn}{dx}$$

By rearranging the above equation, the electric field can be expressed as

$$E\left(x\right)=-\left(\frac{D_{n}-D_{p}}{\mu_{n}+\mu_{p}}\right)\frac{1}{n}\frac{dn}{dx}$$

Using the Einstein relation, *D* = *μk_B_T*/*q* = *μV_T_*, the electric field can be rewritten as

$$E\left(x\right)=\frac{\left(\mu_{p}-\mu_{n}\right)V_{T}}{\mu_{n}+\mu_{p}}\frac{1}{n}\frac{dn}{dx}=V_{T}\frac{\mu_{p}-\mu_{n}}{\mu_{n}+\mu_{p}}\frac{d\left(\ln n\right)}{dx}$$

Furthermore, integrating the electric field along the channel direction *x* gives rise to a lateral potential difference, corresponding to the open-circuit photovoltage V_OC_,

$$V_{OC}=\int_{0}^{L} E\left(x\right)dx=V_{T}\frac{\mu_{p}-\mu_{n}}{\mu_{n}+\mu_{p}}\ln\frac{n\left(L\right)}{n\left(0\right)}$$

**Task 3: Expression for the Short-Circuit Current J_SC_**

Under short-circuit conditions, the electrical potentials at the two terminals are equal,

Based on the drift-diffusion formalism, the short-circuit current density *J_SC_* can be written as

$$J_{SC}=qn\left(\mu_{n}+\mu_{p}\right)E+q\left(\mu_{n}-\mu_{p}\right)V_{T}\frac{dn}{dx}$$

By rearranging the equation and moving the electric field term *E*(*x*) to the left-hand side, one obtains

$$E\left(x\right)=\frac{J_{SC}}{qn\left(\mu_{n}+\mu_{p}\right)}-\frac{\left(\mu_{n}-\mu_{p}\right)V_{T}}{\mu_{n}+\mu_{p}}\frac{1}{n}\frac{dn}{dx}$$

Under the short-circuit boundary condition, the integral of the electric field along the channel vanishes, i.e.,

$$\int_{0}^{L} \left[\frac{J_{SC}}{qn\left(\mu_{n}+\mu_{p}\right)}-\frac{\left(\mu_{n}-\mu_{p}\right)V_{T}}{\mu_{n}+\mu_{p}}\frac{1}{n}\frac{dn}{dx}\right]\text{ }dx=0$$

To simplify the expression, a representative carrier concentration *n_H_* is introduced, which effectively corresponds to the harmonic mean of the spatially varying carrier concentration *n*(*x*) along the channel.

$$\int_{0}^{L} \frac{dx}{n\left(x\right)}\approx\frac{L}{n_{H}}$$

The harmonic mean is defined as

$$H_{n}=\left[\frac{1}{n}\sum_{i=1}^{n} \frac{1}{x_{i}}\right]^{-1}=\frac{n}{\sum_{i=1}^{n} \frac{1}{x_{i}}}\Rightarrow n_{H}=\left[\frac{1}{L}\int_{0}^{L} \frac{1}{n\left(x\right)}dx\right]^{-1}$$

where *H_n_* corresponds to *n_H_*, *n* corresponds to the channel length *L*, and the summation over 1/*x_i_* corresponds the spatial integral over 1/*n*(*x*). Accordingly, substituting *n_H_* into the electric field integral expression yields

$$\begin{matrix} \frac{J{}_{SC}}{q\left(\mu_{n}+\mu_{p}\right)n_{H}}L-\frac{\left(\mu_{n}-\mu_{p}\right)V_{T}}{\mu_{n}+\mu_{p}}\int_{0}^{L} \left[\frac{d\left(\ln n\right)}{dx}\right]\text{ }dx=0 \\ \frac{J{}_{SC}}{q\left(\mu_{n}+\mu_{p}\right)n_{H}}L-\frac{\left(\mu_{n}-\mu_{p}\right)V_{T}}{\mu_{n}+\mu_{p}}\ln\frac{n\left(L\right)}{n\left(0\right)}=0 \end{matrix}$$

Solving for the short-circuit current density *J_sc_* finally gives,

$$J_{SC}=\frac{qn_{H}\left(\mu_{n}-\mu_{p}\right)V_{T}}{L}\ln\frac{n\left(L\right)}{n\left(0\right)}$$

**Task 4: Relation Between the Generation Rates (*G_L_*/*G_R_*) and V_OC_/J_SC_**

Under steady-state conditions, the time derivative of the current vanishes. The inflow and outflow of carriers are balanced with opposite directions, and the recombination rate equals the generation rate. As a result, one can approximately write,

Furthermore, under the low-injection condition, the total carrier concentration can be expressed as the sum of the equilibrium and non-equilibrium contributions, yielding

$$n\left(0\right)\approx n_{i}+G_{L}\tau,\text{ }n\left(L\right)\approx n_{i}+G_{R}\tau$$

Based on Eq. (23), the logarithmic term ln[*n*(*L*)/*n*(0)] appearing in Eqs. (12) and (20) can be rewritten as

$$\ln\left(\frac{n\left(L\right)}{n\left(0\right)}\right)\Rightarrow\ln\left(\frac{n_{i}+G_{R}\tau}{n_{i}+G_{L}\tau}\right)$$

To obtain a more compact expression explicitly related to *G_R_* and *G_L_*, algebraic manipulation can be performed. Under the low-injection condition, *G_R_τ* and *G_L_τ* are smaller than the intrinsic carrier concentration *n_i_*, i.e., *G_R_τ* ≠ *G_L_τ* << *n_i_*. Consequently, the above expression can be rewritten as

$$\ln\left(\frac{n_{i}+G_{R}\tau}{n_{i}+G_{L}\tau}\right)=\ln\left(n_{i}+G_{R}\tau\right)-\ln\left(n_{i}+G_{L}\tau\right)=\ln\left(1+\frac{G_{R}\tau}{n_{i}}\right)-\ln\left(1+\frac{G_{L}\tau}{n_{i}}\right)$$

Using the first-order Taylor expansion ln(1+*x*) ≈ *x* (|*x*| << 1), one obtains

$$\ln\left(\frac{n\left(L\right)}{n\left(0\right)}\right)\approx\frac{G_{R}\tau}{n_{i}}-\frac{G_{L}\tau}{n_{i}}=\frac{\tau}{n_{i}}\left(G_{R}-G_{L}\right)$$

Substituting the above result into the expressions for *V_OC_* and *J_SC_* finally leads to

**Task 5: Implications of the Analytical Model**

Based on the above expressions, several conclusions can be drawn:

1. **Equal generation rates.** When the photocarrier generation rates in the left and right photosensitive regions are equal (*G_L_* = *G_R_*), both the open-circuit voltage *V_OC_* and the short-circuit current *I_SC_* vanish, regardless of whether the electron and hole mobilities are different (*μ_n_* > *μ_p_* or *μ_n_* < *μ_p_*) (Figure S5a, b). In this case, the device exhibits nearly zero photocurrent under uniform illumination.
2. **Interchanged generation rates.** When the generation rates in the left and right photosensitive regions are interchanged (*G_L_* > *G_R_* ↔ *G_L_* > *G_R_*), while the electron mobility remains larger than the hole mobility (*μ_n_* > *μ_p_*), the signs of *V_OC_* and *I_SC_* are reversed (Figure S5c, d). Consequently, the device outputs photocurrents with opposite polarities when detecting left and right edges, respectively.
3. **Reduced generation-rate asymmetry.** When the difference between the generation rates decreases (|*G_L_* - *G_R_*|↓), with *μ_n_* > *μ_p_* fixed, both *V_OC_* and *I_SC_* are reduced in magnitude (Figure S5e, f). This corresponds to a weakening of the photocurrent as the incident optical power decreases.
4. **Equal mobilities.** When the electron and hole mobilities are equal (*μ_n_* = *μ_p_*), both *V_OC_* and *I_SC_* vanish even if the photocarrier generation rates differ between the left and right photosensitive regions (*G_L_* ≠ *G_R_*) (Figure S6a, b).
5. **Interchanged mobilities.** When the electron and hole mobilities are interchanged (μ_n_ > μ_p_ ↔ μ_n_ < μ_p_), while maintaining a higher generation rate in the left photosensitive region (*G_L_* > *G_R_*), the signs of *V_OC_* and *I_SC_* are reversed (Figure S6c, d).
6. **Reduced mobility contrast.** When the difference between the electron and hole mobilities decreases (|μ_n_ - μ_p_|↓), with *G_L_* > *G_R_* fixed, both *V_OC_* and *I_SC_* decrease in magnitude (Figure S6e, f).

**Figure S6. Simulated I-V curves of the CBUP under dark and illuminated conditions, focusing on the effect of photocarrier generation rates.** (a) *G_L_* = *G_R_* with *μ_n_* < *μ_p_*. (b) *G_L_* = *G_R_* with *μ_n_* > *μ_p_*. (c) *G_L_* > *G_R_* with *μ_n_* > *μ_p_*. (d) *G_L_* < *G_R_* with *μ_n_* > *μ_p_*. (e) G_L_ > G_R_ with *μ_n_* > *μ_p_*, where *G_L_* is reduced compared with case (c). (f) *G_L_* < *G_R_* with *μ_n_* > *μ_p_*, where *G_R_* is reduced compared with case (d). The simulated device geometry is identical to that of the experimental device, with the anode on the left and the cathode on the right. The current is defined as positive when flowing from the anode into the device. Symbol definitions: *G_L_*, photocarrier generation rate in the left photosensitive region; *G_R_*, photocarrier generation rate in the right photosensitive region; *μ_n_*, electron mobility; *μ_p_*, hole mobility.

**Figure S7. Simulated I-V curves of the CBUP under dark and illuminated conditions, focusing on the effect of carrier mobilities.** (a) *G_L_* > *G_R_* with *μ_n_* = *μ_p_*. (b) *G_L_* < *G_R_* with *μ_n_* = *μ_p_*. (c) *G_L_* > *G_R_* with *μ_n_* > *μ_p_*. (d) *G_L_* > *G_R_* with *μ_n_* < *μ_p_*. (e) G_L_ > G_R_ with *μ_n_* > *μ_p_*, where *μ_p_* is increased compared with case (c). (f) *G_L_* < *G_R_* with *μ_n_* < *μ_p_*, where *μ_n_* is increased compared with case (d). The simulated device geometry is identical to that of the experimental device, with the anode on the left and the cathode on the right. The current is defined as positive when flowing from the anode into the device. Symbol definitions: *G_L_*, photocarrier generation rate in the left photosensitive region; *G_R_*, photocarrier generation rate in the right photosensitive region; *μ_n_*, electron mobility; *μ_p_*, hole mobility.

**3.3 Photocarrier Generation Mechanisms**

According to the experimental results, the CBUP achieves broadband photodetection at room temperature, with a spectral response spanning from the near-infrared (1.55 μm) to the long-wave infrared (11 μm). The bias-free broadband photoresponse of the CBUP primarily originates from the asymmetric distribution of photogenerated carriers between the left and right photosensitive regions, together with the unequal mobilities of electrons and holes. However, the physical mechanisms responsible for photocarrier generation differ across spectral bands and can be classified into three categories: intrinsic absorption, internal photoemission, and photothermal effects.

**Intrinsic absorption**

In the near-infrared and part of the short-wave infrared region with wavelengths below 1.8 μm, intrinsic absorption dominates the photocarrier generation process. Ge has a bandgap of approximately 0.67 eV, corresponding to a photon wavelength of about 1.8 μm. Incident photons within this spectral range can therefore be directly absorbed by Ge, efficiently generating electron–hole pairs. As a result, the CBUP exhibits its highest responsivity in this wavelength region. In this case, both photogenerated electrons and holes participate in carrier transport, corresponding to bipolar transport, and the steady-state photoresponse can be described using Eqs. 31 and 32.

**Internal photoemission**

In the short-wave to mid-wave infrared region (wavelengths > 1.8 μm), internal photoemission becomes the dominant mechanism. The metallic gratings in the left and right photosensitive regions consist of Ti/Au in contact with Ge. Given that the electron affinity of Ge is 4.13 eV and the work function of Ti is 4.33 eV, a low Schottky barrier of approximately 0.2 eV is formed at the Ti-Ge interface. Such a low barrier allows low-energy infrared photons to excite hot electrons in Ti, enabling them to overcome the barrier and inject into Ge, thereby generating a larger number of carriers in one photosensitive region than the other. Moreover, as reported in Ref. [1], although an Au layer is deposited on top of Ti, more than 57% of the hot electrons are still generated within the Ti layer. Nevertheless, compared with intrinsic absorption, internal photoemission is less efficient, leading to a lower responsivity in this wavelength range.

It is noteworthy that theoretical calculations indicate that a Schottky barrier height of ~0.2 eV corresponds to a photon wavelength of approximately 6.22 μm. Although this wavelength is slightly shorter than the 6.3 μm laser used in the experiments, the corresponding photon energies are very close. Consequently, hot electrons excited under 6.3 μm illumination can still tunnel through the ultrathin interfacial triangular barrier into Ge via quantum tunneling effects[2-3]. In addition, resonant coupling between the metallic grating and the incident infrared light further enhances the hot-electron excitation efficiency. As a result, the CBUP exhibits a commendable photoresponse even under low-energy 6.3 μm illumination. For photons with even lower energies, such as those in the long-wave infrared region, the tunneling probability decreases significantly, leading to a negligible contribution from internal photoemission.

In this regime, the non-equilibrium carriers in the Ge channel predominantly originate from hot-electron injection from the metallic gratings. Under continuous illumination, the spatially non-uniform distribution of injected hot electrons gives rise to diffusion, which in turn establishes an internal electric field through the Poisson equation. This field interacts with the diffusion process until a dynamic steady state is reached. By denoting the hot-electron injection rates in the left and right photosensitive regions as *G_inj,L_* and *G_inj,R_*, respectively, the previous expression in Eq. 32 can be rewritten as

$$J_{SC}\propto\left(\mu_{n}-0\right)\left(G_{inj,R}-G_{inj,\mathrm{L}}\right)$$

As shown in Figure S28, when a mid-wave infrared laser scans across the device from right to left, the hot-electron injection efficiency is initially higher on the right side, resulting in a positive photocurrent, and subsequently becomes higher on the left side, leading to a negative photocurrent. The above expression accurately captures the observed polarity reversal of the photocurrent.

**Photothermal effect**

In the long-wave infrared region, the photothermal effect becomes the dominant mechanism. For example, photons at a wavelength of 8.0 μm have an energy of only ~0.15 eV, which is insufficient for photoexcited hot electrons to overcome the Schottky barrier and inject into Ge. Because the Ge substrate used in the CBUP is intrinsic, impurity-related extrinsic absorption can be excluded. In this case, the incident long-wave infrared radiation interacts with the metallic gratings and Ge channel and is partially converted into heat, resulting in a local temperature rise in the illuminated region. For the intrinsic Ge channel, the carrier concentration is temperature dependent and can be described as,

$$n_{i}\left(T\right)=\sqrt{N_{C}N_{V}}\exp\left(-\frac{E_{g}}{2k_{B}T}\right)$$

where *n_i_*(*T*) is the intrinsic carrier concentration at temperature *T*, *N_C_* and *N_V_* are the effective densities of states in the conduction and valence bands, respectively, *E_g_* is the bandgap, and *k_B_* is the Boltzmann constant. Therefore, a local temperature increase modifies the carrier concentration in the illuminated region, with the carrier concentration generally increasing as the temperature rises.

In the CBUP, the different metallic-grating coverages on the left and right photosensitive regions, or localized long-wave-infrared illumination on one side of the device, can produce an asymmetric lateral temperature distribution, such that the temperature changes in the two photosensitive regions are different (∆*T_L_* ≠ ∆*T_R_*). Correspondingly, the carrier-concentration changes on the two sides are also different. Combined with the static Photo-Dember mechanism discussed above, this lateral carrier-concentration gradient can generate diffusion currents and the corresponding Dember electric field, because electrons and holes have different diffusion capabilities, thereby producing a stable zero-bias photocurrent under short-circuit conditions.

Under the small-temperature-rise approximation, this relationship can be conceptually expressed as,

$$J_{SC}\propto\left(\mu_{n}-\mu_{p}\right)\frac{\partial n}{\partial T}\left(\Delta T_{R}-\Delta T_{L}\right)$$

where *μ_n_* and *μ_p_* are the electron and hole mobilities, respectively, and ∆*T_R_* - ∆*T_L_* represents the effective temperature difference between the right and left photosensitive regions. This expression indicates that the photothermal response follows the same fundamental physical picture as the position-dependent bi-directional photoresponse described in this work. When the locally heated region moves from one side of the device to the other, the lateral temperature gradient and the corresponding carrier-concentration gradient are reversed, resulting in a reversal of the short-circuit photocurrent polarity.

This simplified model also explains the long-wave-infrared response characteristics observed experimentally. Compared with direct interband photoexcitation or internal photoemission, the photothermal response involves optical absorption, thermalization, temperature establishment, and subsequent thermal relaxation. Consequently, the response is generally weaker and occurs on a longer timescale. The experimentally observed reduction in long-wave-infrared response amplitude and slower response speed are consistent with this photothermal-dominated physical picture.

**Overall picture**

In summary, although the physical mechanisms responsible for generating non-equilibrium carriers differ across spectral bands, they all lead to a spatially non-uniform distribution of photocarriers in the CBUP. The overall operation of the device can be summarized as follows: non-uniform generation → diffusion → space-charge formation → internal electric field → drift-diffusion balance → steady-state photoresponse. This sequence persists as long as illumination is present, and the device returns to its dark steady state once the incident light is removed.

**3.4 Summary of Symbols, Assumptions, and Boundary Conditions**

**Table S1. Symbols, Assumptions, and Boundary Conditions used in Section 5**

| **Part 1. Symbols and definitions** | |
| --- | --- |
| **Symbol** | **Definition** |
| *x* ∈ [0, *L*] | Spatial coordinate along the Ge channel |
| *L* | Channel length |
| *n*(*x*), *p*(*x*) | Electron concentration, Hole concentration |
| *n_i_* | Intrinsic carrier concentration of Ge |
| *φ*(*x*) | Electrostatic potential |
| *E*(*x*) | Electric field, defined as *E*(*x*) = -d*φ*/d*x* |
| J_n_(x) | Electron current density |
| J_p_(x) | Hole current density |
| *G_L_*, *G_R_* | Effective photocarrier generation rates in the left and right photosensitive regions (cm^-3^·s^-1^) |
| *G_inj,L_*, *G_inj,R_* | Effective hot-electron injection rates in the left and right regions (sub-bandgap regime) |
| *μ_n_*, *μ_p_* | Electron mobility, Hole mobility |
| *D_n_* | Electron diffusion coefficient, *D_n_* = *μ_n_V_T_* |
| *D_p_* | Hole diffusion coefficient, *D_p_* = *μ_p_V_T_* |
| *V_T_* | Thermal voltage, *V_T_* = *k_B_T*/*q* |
| *τ* | Effective carrier lifetime |
| ∆*n*(*x*) | Excess carrier concentration under illumination |
| *n_L_*, *n_R_* | Representative carrier concentrations in the left and right photosensitive regions |
| Low-injection approximation:  Under local steady-state and low-injection conditions, ∆*n*(*x*) ≈ *G*(*x*)*τ*, *n*(*x*) ≈ *n_i_* + ∆*n*(*x*),  and for the left/right regions, *n_L_* ≈ *n_i_* + *G_L_τ*, *n_R_* ≈ *n_i_* + *G_R_τ* | |
| **Part 2. Boundary conditions and operating assumptions** | |
| **Condition** | **Description** |
| Device geometry | One-dimensional intrinsic Ge channel with symmetric Ohmic contacts |
| Electrode definition | Left electrode: Anode; right electrode: Cathode |
| Current convention | Current flowing from the anode into the channel is defined as positive |
| Open-circuit condition | External circuit open; total current *J* = 0 |
| Open-circuit voltage | Terminal potentials are allowed to differ: *V_OC_* = *φ*(*L*) – *φ*(0) ≠ 0 |
| Short-circuit condition | Terminals shorted by an ideal conductor |
| Short-circuit boundary | *φ*(0) = *φ*(*L*), leading to $\int_{0}^{L} E(x)\text{ }dx=0$ |

In numerical simulations, the electron and hole concentrations in the vicinity of Ohmic contacts are driven toward values close to local thermal equilibrium (see Figure S11d). This is a direct consequence of the default Ohmic-contact boundary condition, under which the contact behaves as an ideal carrier reservoir: the electrostatic potential is prescribed by the applied terminal voltage (0 V in this work), and the carrier densities at the contact nodes are pinned near their equilibrium values set by the local doping. As a result, excess non-equilibrium carriers generated in the device bulk are collected at the contacts, and the associated carrier flux appears as the terminal current through the electrodes. Consistent with this “reservoir” behaviour, the net space-charge density becomes negligible at the Ohmic-contact nodes (see Figure S7), even though a pronounced, illumination-induced space-charge asymmetry persists inside the active region.

In contrast, the analytical derivation of *J_SC_* (see Eq. 28) presented here does not treat the contacts as ideal reservoirs that enforce equilibrium carrier densities exactly at the endpoints. Instead, generation-recombination processes are incorporated through an effective bulk carrier lifetime, and the model focuses on the steady-state photoresponse arising from non-uniform photocarrier generation and the mobility mismatch between electrons and holes. In simple terms, the analytical model evaluates $n$at the inner edge of the contact region rather than at the contact node. Consequently, *n*(0^+^) and *n*(*L*^-^) can differ, consistent with the asymmetric carrier-density gradients on the two sides observed in Figure S11d. Therefore, despite differences in the detailed boundary-condition treatment between the analytical model and the numerical simulations, both approaches yield consistent dependencies of *V_OC_* and *J_SC_* on the photocarrier generation rates (*G_R_*/*G_L_*) and the mobilities (*μ_n_*/*μ_p_*).

It should be emphasised that a more rigorous steady-state analytical description can still be formulated within the drift-diffusion framework. However, such a treatment must take into account the different distributions of *n*(*x*) and *p*(*x*) under illumination, particularly under asymmetric spatial photocarrier generation and unequal electron/hole mobilities. As illustrated in Figure S7, a non-vanishing and spatially asymmetric space-charge distribution is observed. Consequently, one cannot, as in Eq. (8), assume *n*(*x*) = *p*(*x*) everywhere and factor them out of the relevant terms; instead, Eq. (8) must be generalised to Eq. (30).

$$\begin{matrix} J=q\left[n\left(x\right)\mu_{n}+p\left(x\right)\mu_{p}\right]E+qV_{T}\left[\mu_{n}\frac{dn\left(x\right)}{dx}-\mu_{p}\frac{dp\left(x\right)}{dx}\right] \\ n\left(x\right)=n_{i}+\Delta n\left(x\right),\text{ }p\left(x\right)=n_{i}+\Delta p\left(x\right) \end{matrix}$$

The subsequent derivation follows the same procedure as before, and the boundary conditions can likewise be extended to the endpoints *x* = 0 and *x* = *L*. Nevertheless, because it is generally difficult to obtain closed-form expressions for the full spatial profiles *n*(*x*) and *p*(*x*), the approximation *n*(*x*) ≈ *p*(*x*) in the main derivation is adopted to yield a more compact and transparent expression. This simplified form highlights the explicit relationships between *V_OC_* (or *J_SC_*), the left/right photocarrier generation rates, and the electron/hole mobilities, thereby clarifying their interplay and facilitating interpretation of both the simulations and the experimental observations.

**Figure S8. Simulated space charge distribution in the Ge channel under illumination.**

**3.5 Definition and Scope of the Terminology**

In this work, “computation-less” specifically refers to device-level spatial differentiation: the CBUP directly converts a local optical-intensity gradient into a signed photocurrent through its intrinsic position-dependent bi-directional photoresponse, without requiring numerical convolution to generate the elementary edge response. Signal readout, absolute-value processing, normalization, grayscale mapping, multidirectional gradient fusion, and image visualization still require peripheral circuitry or subsequent data processing. Therefore, “computation-less” in this work does not imply a completely computation-free system-level workflow.

Here, “bias-free” refers specifically to the absence of an externally applied voltage across the active region of the CBUP during photodetection. It does not imply zero power consumption at the system level, because array addressing, signal readout, amplification, and subsequent processing may still require external power.

Moreover, “single-pixel edge extraction” refers specifically to local first-order spatial-gradient sensing by an individual CBUP along its OEDV direction. When the local optical-intensity distribution contains a pronounced spatial transition along this direction, the position-dependent bi-directional photoresponse of the device directly converts the local intensity gradient into a signed photocurrent. In contrast, for an approximately uniform optical-intensity distribution with a near-zero local gradient, the responses from the two photosensitive regions tend to compensate each other, resulting in a near-zero output.

- **Section 4. Position-dependent Bi-directional Photoresponse**

Figure S8a illustrates the internal light intensity distribution when the incident spot is positioned at different locations on the device surface. From stage I to stage VIII, the light spot moves from the cathode toward the anode. For the CBUP with an OEDV of [-1, 0, 1] (Figure S8b), when the light spot is located in the left photosensitive region, the photocarrier generation rate in this region is high, resulting in a negative photocurrent (NPC). When the light spot is located at the device center, the photocarrier generation rates of the photosensitive regions on the left and right sides are nearly equal, and the photocurrent approaches zero (ZPC). As the light spot continues to move to the right, the photocarrier generation rate in the right region increases, leading to a positive photocurrent (PPC). The CBUP with an OEDV of [-1.2, 0, 1] also exhibits a position-dependent bi-directional photoresponse. However, due to the asymmetric metallic gratings on both sides of the channel, the magnitude of the minimum negative photocurrent is larger than that of the maximum positive photocurrent.

The physical models used during the TCAD simulation include Fermi-Dirac statistics, Poisson distribution, continuity formulation, drift-diffusion equation, Shockley-Read-Hall recombination, and optical excitation. The doping of Ge channel was assumed to be 0 cm^-3^ for intrinsic doping. The contact between the electrode and the Ge channel was set as ohmic contact.

**Figure S9. Position-dependent bi-directional photoresponse.** (a) Trajectory of the moving light spot in the simulation. (b) Simulated photocurrent of the CBUP with an OEDV of [-1, 0, 1] under different illumination powers as a function of the light spot position. The simulations were performed with a light source of 1.55 μm wavelength, and a bias of 0 V. The curve corresponding to maximum power in (b) is reused in Figure 1e.

To examine whether the position-dependent bi-directional photoresponse can be extended from Ge to Si, additional device simulations were performed using a Si channel. The device geometry and boundary conditions were kept consistent with those used for the Ge-based CBUP, except that the channel material was replaced by Si.

As shown in Figure S9, when the light spot is located on the right side, near the center, and on the left side of the Si channel, the simulated short-circuit current exhibits positive, near-zero, and negative outputs, respectively. The reversal of the current polarity as the light-spot position moves from one side of the channel to the other demonstrates that the position-dependent bi-directional photoresponse can also be preserved in a Si-based device.

**Figure S10. Position-dependent bi-directional photoresponse of the Si-based CBUP.** (a-i) Simulated optical intensity distribution and (a-ii) Simulated I-V curve, where the light spot is located in the left photosensitive region. (b-i) Simulated optical intensity distribution and (b-ii) Simulated I-V curve, where the light spot is located in the center of the channel. (c-i) Simulated optical intensity distribution and (c-ii) Simulated I-V curve, where light spot is located in right photosensitive region.

This behavior is consistent with the underlying transport mechanism discussed in Section 3. In addition to a spatially nonuniform photocarrier-generation profile, the mechanism requires asymmetric electron and hole transport. Because Si also exhibits different electron and hole mobilities, the resulting differential diffusion can establish a lateral Photo-Dember field and generate a position-dependent short-circuit current under suitable boundary conditions. These results indicate that the physical mechanism underlying the CBUP is not restricted to Ge and may provide a pathway toward Si-based implementations. Since Si is the foundational semiconductor of CMOS technology, such a Si-based implementation may further facilitate future exploration of CMOS-compatible integration.

- **Section 5. Symmetry-enabled Bias-free Photodetection**

The photoresponse of the photoconductor with an OEDV of [-1, 0, +1] under zero bias and uniform illumination is illustrated in Figure S10. The device consists of a uniformly undoped Ge channel with metal electrodes on both sides (Figure S10a), where ohmic contacts are set between the electrodes and the channel. Under uniform illumination, the generation rate of photocarriers remains constant throughout the channel (*G_L_* = *G_R_*, Figure S10b). As shown in the spatial distribution of electron density (Figure S10c, d), the distribution is symmetric with respect to the channel center, and the migration vectors of electrons point symmetrically from the center toward both electrodes, leading to a photocurrent close to zero (Figure S10e).

**Figure S11. Photoresponse analysis of a photoconductor with an OEDV of [-1, 0, +1].** (a) Schematic diagram of the device structure. (b) Simulated optical generation along the horizontal dashed line in the channel. (c) Simulated electron density distribution, with arrows indicating the direction of electron migration. (d) Electron density profile along the horizontal dashed line. (e) Simulated I-T curve at 0 V bias. The wavelength of incident light is 1.55 μm.

The photoresponse of the CBUP with an OEDV of [-1.2, 0, +1] under zero bias and uniform illumination is shown in Figure S11. The two photosensitive regions of the CBUP adopt different grating designs (Figure S11a), resulting in distinct photocarrier generation rates on the left and right sides (*G_L_* > *G_R_*, Figure S11b). Specifically, the left photosensitive region has a smaller grating coverage than the right one, and the light-metal grating interaction in this region is weaker, leading to higher transmittance and thus more photocarriers. As illustrated in Figure S11c, d, the electron density distribution in the CBUP is no longer symmetric about the channel center but is shifted toward the left side, which leads to an NPC (Figure S11e). This result is consistent with experimental observations.

**Figure S12. Photoresponse analysis of the CBUP with an OEDV of [-1.2, 0, +1].** (a) Schematic diagram of the device structure. (b) Simulated optical generation along the horizontal dashed line in the channel. (c) Simulated electron density distribution, with arrows indicating the direction of electron migration. (d) Electron density profile along the horizontal dashed line. (e) Simulated I-T curve at 0 V bias. The wavelength of incident light is 1.55 μm.

- **Section 6. Edge Extraction Based on CBUPs with Different OEDVs**

Figure S12 illustrates the mechanism of computation-less edge extraction enabled by the CBUP. Benefiting from its position-dependent bi-directional photoresponse, the CBUP can generate directionally distinguishable photocurrents in response to the light intensity gradients at target edges. For a CBUP with an OEDV of [-1, 0, 1], when illuminated by the right (left) edge of a target, a large number of photocarriers are generated on the right (left) side of the channel, producing a PPC (NPC). Under uniform illumination or in darkness, the CBUP outputs ZPC.

The CBUP with an OEDV of [-1.2, 0, 1] via asymmetric metallic grating design exhibits a similar behavior, it also generates a PPC for the right edge and an NPC for the left edge, while maintaining a ZPC in the dark. However, due to the asymmetric photoresponse weights, the maximum PPC is slightly weaker than the minimum NPC. Notably, even under uniform illumination, the CBUP with an OEDV of [-1.2, 0, 1] produces a non-zero NPC, which allows the device to retain partial information from non-edge region of the target and enhances the perception of internal texture features (see Figure 4 in the main text).

**Figure S13. Illustration of computation-less edge extraction based on CBUPs.** (a, b) Schematic diagrams of edge extraction mechanisms for CBUPs with OEDVs of [-1, 0, 1] and [-1.2, 0, 1], respectively. The electron density distributions are obtained from simulations. The electron distribution in (a) is reused in Figure 1f.

- **Section 7. CBUPs with Different Grating Coverage Ratios**

By adjusting the distance between the microscope objective and the CBUP to gradually defocus, a 1.55 μm laser spot with a diameter of approximately 50 μm can be obtained on the device surface, which is used to analyze the photoresponse of the left and right photosensitive regions of the CBUP. As shown in Figures S13-19, when the spot is located in the left photosensitive region, the CBUP generates an NPC, whereas a PPC is observed when the light spot is positioned in the right region.

Furthermore, by adjusting the grating coverage ratio in the left and right photosensitive regions, the positive-to-negative photoresponse ratio of the CBUP can be controlled. Importantly, the numerical values of the corresponding OEDV can be approximately determined from the experimentally measured positive and negative photocurrents when the illumination spot is positioned over the two opposite photosensitive regions. For example, for the CBUP with a left-to-right grating-coverage ratio of 2:3, the measured ratio between the magnitudes of the negative and positive photocurrents is ~1.228. By normalizing the smaller response magnitude to 1 and rounding the ratio to one decimal place for simplified modelling, the corresponding OEDV is represented as [-1.2, 0, +1].

**Figure S14. CBUP with grating coverage of 3:3 in the left and right photosensitive regions.** (a) Top panel: Optical microscope image of the CBUP; Bottom panel: Dark-field microscope image of the CBUP. The dark-blue (red) dashed circle marks the position of the 1.55 μm laser spot on the left (right) photosensitive region. Scale bar: 10 μm. (b, c) Measured I-T curves of the CBUP under zero bias when the laser spot is located in the left and right photosensitive regions, respectively.

**Figure S15. CBUP with grating coverage of 2:3 in the left and right photosensitive regions.** (a) Top panel: Optical microscope image of the CBUP; Bottom panel: Dark-field microscope image of the CBUP. The dark-blue (red) dashed circle marks the position of the 1.55 μm laser spot on the left (right) photosensitive region. Scale bar: 10 μm. (b, c) Measured I-T curves of the CBUP under zero bias when the laser spot is located in the left and right photosensitive regions, respectively.

**Figure S16. CBUP with grating coverage of 1:3 in the left and right photosensitive regions.** (a) Top panel: Optical microscope image of the CBUP; Bottom panel: Dark-field microscope image of the CBUP. The dark-blue (red) dashed circle marks the position of the 1.55 μm laser spot on the left (right) photosensitive region. Scale bar: 10 μm. (b, c) Measured I-T curves of the CBUP under zero bias when the laser spot is located in the left and right photosensitive regions, respectively.

**Figure S17. CBUP with grating coverage of 0:3 in the left and right photosensitive regions.** (a) Top panel: Optical microscope image of the CBUP; Bottom panel: Dark-field microscope image of the CBUP. The dark-blue (red) dashed circle marks the position of the 1.55 μm laser spot on the left (right) photosensitive region. Scale bar: 10 μm. (b, c) Measured I-T curves of the CBUP under zero bias when the laser spot is located in the left and right photosensitive regions, respectively.

**Figure S18. CBUP with grating coverage of 3:2 in the left and right photosensitive regions.** (a) Top panel: Optical microscope image of the CBUP; Bottom panel: Dark-field microscope image of the CBUP. The dark-blue (red) dashed circle marks the position of the 1.55 μm laser spot on the left (right) photosensitive region. Scale bar: 10 μm. (b, c) Measured I-T curves of the CBUP under zero bias when the laser spot is located in the left and right photosensitive regions, respectively.

**Figure S19. CBUP with grating coverage of 3:1 in the left and right photosensitive regions.** (a) Top panel: Optical microscope image of the CBUP; Bottom panel: Dark-field microscope image of the CBUP. The dark-blue (red) dashed circle marks the position of the 1.55 μm laser spot on the left (right) photosensitive region. Scale bar: 10 μm. (b, c) Measured I-T curves of the CBUP under zero bias when the laser spot is located in the left and right photosensitive regions, respectively.

**Figure S20. CBUP with grating coverage of 3:0 in the left and right photosensitive regions.** (a) Top panel: Optical microscope image of the CBUP; Bottom panel: Dark-field microscope image of the CBUP. The dark-blue (red) dashed circle marks the position of the 1.55 μm laser spot on the left (right) photosensitive region. Scale bar: 10 μm. (b, c) Measured I-T curves of the CBUP under zero bias when the laser spot is located in the left and right photosensitive regions, respectively.

Because the metallic gratings used to engineer the spatial photoresponse may exhibit polarization dependence, variations in the incident polarization state can potentially modulate the edge-response amplitude. To evaluate this effect, the polarization-dependent photoresponse of the CBUP was measured under 1.55 μm illumination with the optical spot positioned over the left photosensitive region. As shown in Figure S20, the photocurrent remains negative over the entire measured polarization-angle range, while the ratio between the maximum and minimum response magnitudes is ~1.06. These results indicate that polarization variation mainly introduces a limited modulation of the response amplitude under the present test conditions without reversing the photocurrent polarity. Such weak polarization dependence is beneficial for the present edge-sensing function because it reduces the risk that changes in the incident polarization state alter the sign of the edge response.

**Figure S21. Polarization-dependent photocurrent of the CBUP.** Measured Current as a function of the incident polarization angle when the 1.55 μm laser spot is positioned on the left photosensitive region. The selected CBUP has a left-to-right grating coverage ratio of 2:3, and the applied bias is 0 V.

- **Section 8. Performance Summary**

**Table S2. Performance Summary of Reported Photoconductors.**

| **No.** | **Description** | **Wavelength (μm)** | **On-off ratio** | **Optical power** | **Bias Voltage** | **Ref.** | **Remarks** |
| --- | --- | --- | --- | --- | --- | --- | --- |
| 1 | Ultra-thin Ge | Vis - NIR | ~ 52.5 | 2.45 μW | 1 V | [4] | / |
| 2 | Ge nanowire | 0.532  (Vis) | ~ 1.9 | 32 nW | 2 V | [5] | / |
| 3 | Perovskite/Ge | 0.405-1.55  (Vis - NIR) | ~ 10 | 1.94 mW | 1 V | [6] | / |
| 5 | Ge/Si | 1.55  (NIR) | ~ 1.5 | 468 μW | 1 V | [7] | / |
| 6 | Gra/Si Grating | 1.55 - 2.7  (NIR - SWIR) | ~ 1.4 | 144 μW | 3 V | [8] | / |
| 7 | Ti/Gra/Pd | 1.55  (NIR) | ~ 1.2 | 20 dBm | 0.1 V | [9] | / |
| 8 | Pd-MoS_2_/Gra-Ti | 0.632  (Vis) | 1.11 | 0.82 μW | 0.1 V | [10] | / |
| 9 | Te nanowires | 0.5 - 2.5  (Vis - SWIR) | ~ 1.75 | 250 μW | 1 V | [11] | Blackbody sensitivity |
| 10 | Gra/Al_2_O_3_/LiNbO_3_ | 8 - 11  (LWIR) | ~ 2.9 | 139 nW | 0.2 V | [12] | Blackbody sensitivity |
| 11 | Gra/Ge | 0.635 - 1.55  (Vis - NIR) | ~ 1.35 | 373 nW | 1 V | [13] | Blackbody sensitivity and edge detection |
| 12 | WSe_2_/h-BN/Al_2_O_3_ | 0.405  Vis | ~ 3.1 | 4.2 nW/μm^2^ | 10 mV | [14] | Edge detection |
| 13 | HfO_2_/Al_2_O_3_/bP | 1.55  (NIR) | ~ 1.6 | 10 mW | 100 mV | [15] | Edge detection |
| 14 | MoS_2_/SiN_x_/Si | 0.385 - 0.98  (Vis - NIR) | 10 | 350 nW | 0.1 V | [16] | Edge detection |
| 15 | Perovskite/Gra/Ge | 0.375-1.55  (UV - NIR) | ~ 1.6 | 2 μW | 1 V | [17] | Functional |
| 16 | Asymmetric metallic grating + Ge | 1.55 - 11.0  (NIR - LWIR) | 3×10^4^ | 1.46 μW | 0 V | This work | Bias free, blackbody sensitivity, and edge detection |

High-performance photoconductors operating around the near-infrared spectral range have employed a variety of approaches to enhance responsivity and suppress dark current. Trap engineering can increase photoconductive gain by extending the lifetime of photocarriers[18], while heterojunction engineering can facilitate carrier separation and transport through band alignment and interfacial electric fields[19-20]. Plasmonic and optical-field-enhancement structures have also been introduced to improve optical absorption[21-22]. In addition, heterogeneous integration of a photosensitive layer with a thin transport channel can combine interfacial depletion and photovoltage modulation to achieve enhanced photoresponse together with reduced dark current[23-24]. These strategies have enabled favorable photo-to-dark-current ratios in recently reported photoconductors. Accordingly, compared with these studies, the distinguishing feature of the CBUP is the combination of a relatively high light-to-dark output contrast with a simple two-terminal structure, zero external bias across the active region during operation, a position-dependent bi-directional photoresponse, and intrinsic device-level edge sensitivity.”

**Table S3. Performance Summary of Typical Photoemissive Infrared Photodetectors.**

| **No.** | **Description** | **Wavelength (μm)** | **Responsivity**  **(mA/W)** | **EQE**  **(%)** | **Bias Voltage** | **Working temperature** | **Ref.** |
| --- | --- | --- | --- | --- | --- | --- | --- |
| 1 | PtSi/p-Si  (φ_b_ ≈ 0.225 eV) | 0.4 - 5.2  (Vis - MWIR) | 30  (@3.0 μm) | 1.243  (@3.0 μm) | 10 V | 77 K | [25] |
| 2 | PtSi/p-Si  (φ_b_ ≈ 0.22 eV) | 2.5 - 6.0  (SWIR - MWIR) | 22.6  (@4.0 μm) | 0.702  (@4.0 μm) | 5 V | 80 K | [26] |
| 3 | Pd_2_Si/p-Si  (φ_b_ ≈ 0.34 eV) | 1.0 - 3.5  (NIR - SWIR) | 16  (@2.5 μm) | 0.795  (@2.5 μm) | 10 V | 40 - 145 K | [27] |
| 4 | IrSi/p-Si  (φ_b_ ≈ 0.094 eV) | 2.0 - 12.4  SWIR-LWIR | 0.8  (@11.0 μm) | 0.009  (@11.0 μm) | 1 V | 40 K | [28] |
| 5 | IrSi/p-Si  (φ_b_ ≈ 0.151 eV) | 2.0 - 8.2  SWIR-LWIR | 0.2  (@7.0 μm) | 0.004  (@7.0 μm) | 5 V | 70 K | [29] |
| 6 | Au/Ti/p-Si Grating  (φ_b_ ≈ 0.44 eV) | 1.0 - 1.6  (NIR) | 51.2  (@1.45 μm) | 4.390  (@1.45 μm) | 1.5 V | 300 K | [1] |
| 7 | Gra/p-Si  (φ_b_ ≈ 0.61 eV) | NIR | 0.16  (@2.0 μm) | 0.010  (@2.0 μm) | 2 V | 300 K | [30] |
| 8 | Au antenna/n-Si  (φ_b_ ≈ 0.5 eV) | 1.2 - 2.5  NIR - SWIR | 0.01  (@1.25 μm) | 0.001  (@1.25 μm) | 0 V | 300 K | [31] |
| 9 | Al/p-Si pyramid  (φ_b_ ≈ 0.6 eV) | NIR | 12  (@1.3 μm) | 1.148  (@1.3 μm) | 1 V | 300 K | [32] |
| 10 | Asymmetry metallic grating + i-Ge  (φ_b_ ≈ 0.2 eV) | 1.55 - 11.0  (NIR - LWIR) | 390 (@1.55 μm)  35 (@2.7 μm)  0.72 (@6.3 μm)  0.25 (@8.0 μm) | 31.28 (@1.55 μm)  1.612 (@2.7 μm)  0.014 (@6.3 μm)  0.004 (@8.0 μm) | 0 V | 300 K | This work |

- **Section 9. NIR Photoresponse of the CBUP**

**Figure S22. NIR photoresponse of the CBUP.** (a, b) Measured I-V curves of the CBUP measured in the dark and under 1.55 μm laser illumination, with the horizontal axis plotted in linear and logarithmic scales, respectively. The dark I-V curve exhibits good linearity, indicating the formation of ohmic contact between the electrodes and Ge. Under 1.55 μm laser illumination, the I-V curve shifts, giving rise to a short-circuit current (I_SC_) and an open-circuit voltage (V_OC_). According to Eqs. 27 and 28, since the light spot is located in the left photosensitive region, negative I_SC_ and positive V_OC_ can be obtained.

The rise and decay times of the CBUP are in the sub-millisecond range, indicating that the measured photoresponse speed is, in terms of timescale, compatible with video-rate imaging. It should be noted that the actual frame rate of a device array would depend not only on the device response time, but also on the integration time, array size, pixel-addressing scheme, analogue-to-digital conversion speed, and data-output bandwidth. Therefore, the current results mainly indicate that the measured device response time is not expected to be a primary limitation for operation at standard video frame rates. To improve the response speed, reducing the channel length could be a feasible approach, as it could shorten the time required for photogenerated carriers to establish a stable spatial distribution and generate a stable photocurrent output.

**Figure S23. Transient photoresponse of the CBUP.** (a) Measured time-resolved current of the CBUP under 1.55 μm laser illumination at voltage = 0 V. (b) Enlarged view of the red boxes in (a), showing the rise and fall times. (c) Measured time-resolved current of the CBUP under 1.55 μm laser illumination at voltage = 10 μV. (d) Enlarged view of the red boxes in (c), showing the rise and fall times. (e) Measured time-resolved current of the CBUP under 1.55 μm laser illumination at voltage = 100 μV. (f) Enlarged view of the red boxes in (e), showing the rise and fall times.

- **Section 10. SWIR Photoresponse of the CBUP**

**Figure S24. Measured I-V curves of the CBUP under 2.7 μm laser illumination.** As described in the main text, the beam was aligned such that its center was positioned approximately over the left photosensitive region to obtain a negative photocurrent response. The spot diameter of the 2.7 μm laser was approximately 1 mm.

**Figure S25. Photoresponse of the CBUP at 2.7 μm.** (a-c) Calculated responsivity (R), noise-equivalent power (NEP), and specific detectivity (D^*^) as a function of incident optical power. Bias voltage is 0 V.

- **Section 11. MWIR Photoresponse of the CBUP**

**Figure S26. Measured I-V curves of the CBUP under MWIR illumination at 3.7 μm.** The inset shows the corresponding curves plotted on a linear scale. As described in the main text, the beam was aligned such that its center was positioned approximately over the left photosensitive region to obtain a negative photocurrent response. The spot diameters of the 3.7 and 6.3 μm lasers were approximately 2 mm.

**Figure S27. Photodetection performance of the CBUP at 3.7 μm.** (a) Measured I-T curves of the CBUP under different illumination powers at 0 V bias. (b-d) Calculated absolute value of photocurrent, responsivity, specific detectivity (D^*^), and noise-equivalent power (NEP) as functions of incident optical power.

**Figure S28. Photodetection performance of the CBUP at 6.3 μm.** (a) Measured I-T curves of the CBUP under different illumination powers at 0 V bias. (b-d) Calculated absolute value of photocurrent, responsivity, specific detectivity (D^*^), and noise-equivalent power (NEP) as functions of incident optical power.

- **Section 12. Position-Dependent WMIR Bi-directional Photoresponse**

Figure S28 shows the output current signals of the CBUP under back-and-forth scanning with 3.7 μm and 6.3 μm laser spots. As the spot moves across the CBUP, the output signal transitions sequentially from positive photocurrent to zero and then to negative photocurrent; reversing the scanning direction correspondingly inverts the photocurrent polarity. These results are consistent with the position-dependent bi-directional photoresponse of the CBUP.

It is noteworthy that the minimum negative photocurrent exhibits a slightly smaller absolute value than the maximum positive photocurrent. This can be attributed to a fact that this device possesses a grating coverage of 2:3 (see Figure S14). Therefore, the left photosensitive region contains fewer metal gratings than the right, resulting in more hot-electron generation on the right side and hence a stronger positive photoresponse.

**Figure S29. Position-dependent photoresponse of the CBUP.** (a, b) Measured I-T curves of the CBUP under back-and-forth scanning of the laser spot. The wavelength of the incident light is 3.7 μm and 6.3 μm, respectively. NPC, ZPC, and PPC denote negative, zero, and positive photocurrent, respectively.

In contrast, although the 1.55 μm laser can also excite hot electrons in the metal, the intrinsic absorption of Ge dominates at this wavelength, and the gratings mainly act as reflectors and transmitters. Consequently, the left photosensitive region, with lower grating coverage, exhibits higher optical transmittance, leading to stronger intrinsic absorption in Ge and thus a negative photoresponse that surpasses the positive one under 1.55 μm illumination.

Taking Figure S28a as an example, the on/off states and position shifts of the laser spot are illustrated in Figure S29. The entire process can be divided into five phases (Figure S29a). In Phases 1, 3, and 5, the laser spot remains fixed in position and only switches between on and off states; in Phases 2 and 4, the laser remains on while moving between the two photosensitive regions. In Phase 1, the spot is located in the right photosensitive region (*G_L_* < *G_R_*), producing a macroscopic current flowing from the anode to the cathode, i.e., a PPC (Figure S29b). In Phase 2, the spot first moves away from the right side and then shifts to the left photosensitive region (Figure S29c), causing the photocurrent to gradually decrease from positive, cross zero, and become negative. In Phase 3, the spot resides in the left photosensitive region (*G_L_* > *G_R_*), generating a macroscopic current flowing from the cathode to the anode, i.e., an NPC (Figure S29d). In Phase 4, the spot moves away from the left side and returns to the right photosensitive region (Figure S29e), leading the current to weaken from negative, cross zero, and recover to be positive. Since the spot remains on the right photosensitive region, the photocurrent polarity in Phase 5 is consistent with that in Phase 1 (Figure S29f).

**Figure S30. Schematic illustration of the laser spot scanning.** (a) Illustration of the laser spot states across five phases. (b-f) Corresponding on/off states and position trajectories of the laser spot for Phases 1-5.

- **Section 13. LWIR Photoresponse of the CBUP**

**Figure S31. Photodetection performance of the CBUP at 8.0 μm.** (a) Measured I-T curves of the CBUP under different illumination powers at 0 V bias. (b-d) Calculated absolute value of photocurrent, responsivity, specific detectivity (D^*^), and noise-equivalent power (NEP) as functions of incident optical power. As described in the main text, the beam was aligned such that its center was positioned approximately over the left photosensitive region to obtain a negative photocurrent response. The spot diameters of the 8.0-11.0 μm lasers were approximately 2.5 mm.

**Figure S32. Photodetection performance of the CBUP at 9.0 μm.** (a) Measured I-T curves of the CBUP under different illumination powers at 0 V bias. (b-d) Calculated absolute value of photocurrent, responsivity, specific detectivity (D^*^), and noise-equivalent power (NEP) as functions of incident optical power.

**Figure S33. Photodetection performance of the CBUP at 10.0 μm.** (a) Measured I-T curves of the CBUP under different illumination powers at 0 V bias. (b-d) Calculated absolute value of photocurrent, responsivity, specific detectivity (D^*^), and noise-equivalent power (NEP) as functions of incident optical power.

**Figure S34. Photodetection performance of the CBUP at 11.0 μm.** (a) Measured I-T curves of the CBUP under different illumination powers at 0 V bias. (b-d) Calculated absolute value of photocurrent, responsivity, specific detectivity (D^*^), and noise-equivalent power (NEP) as functions of incident optical power.

- **Section 14. Blackbody Radiation Response Measurement**

Figure S34a illustrates the schematic of the system used to measure the blackbody response of the CBUP. Under ambient conditions at room temperature, the radiance *L* of an ideal blackbody at temperature *T* can be calculated using the Stefan-Boltzmann law,

$$L=\frac{\sigma\left(T^{4}-T_{0}^{4}\right)}{\pi}$$

where *σ* is the Stefan-Boltzmann constant and *T*_0_ is the room temperature (300 K). As shown in Figure S34b, with increasing blackbody temperature, the radiated power increases and the peak wavelength shifts toward shorter wavelengths.

Furthermore, based on radiometric principles, the power incident on the photosensitive area of the CBUP can be expressed as:

$$\Phi_{d}=\frac{\sigma A_{S}A_{d}}{\pi r^{2}}\times\left(T^{4}-T_{0}^{4}\right)$$

where *Φ_d_* is the radiant flux, *A_s_* is the blackbody aperture area, *A_d_* is the device photosensitive area, and *r* is the distance between the CBUP and the blackbody aperture. The corresponding calculated results are shown in Figure S34c.

**Figure S35. Blackbody radiation measurement and theoretical calculation.** (a) Schematic of the blackbody radiation response measurement setup. (b) Calculated spectral distribution of blackbody radiation at different temperatures. The dashed lines indicate the trend of peak wavelength shift with temperature. (c) Calculated blackbody radiative power incident on the device surface based on radiance formulas.

Figure S35 shows the I-V curves of the CBUP under different blackbody temperatures. As the temperature increases from 850 K to 1100 K, the device exhibits enhanced photocurrent.

**Figure S36. Measured I-V curves of the CBUP under blackbody radiation at different temperatures.** (a-f) Blackbody temperatures of 950 K, 1000 K, 1050 K, and 1100 K, respectively. Insets show the I-V curves plotted on linear scales.

To calculate the specific detectivity (*D**) and noise-equivalent power (*NEP*) of the CBUP, their definitions were used:

$$D^{*}=R\times\frac{\sqrt{A_{d}\cdot\Delta f}}{i_{n}},\text{ }NEP=\frac{i_{n}}{R}$$

where *R* is the responsivity, ∆*f* is the electrical bandwidth (∆*f* = 1 Hz), *A_d_* is the effective photosensitive area of the device, and *i_n_* is the noise current. The noise value measured at a frequency of 1 kHz in the noise spectrum was used for the calculation.

Because the illumination area of the blackbody source was substantially larger than the device channel, a metal-covered region was fabricated around the channel to minimize the absorption of blackbody radiation and the generation of photocarriers in the surrounding non-channel regions. This design suppresses the contribution of the non-channel regions to the measured photoresponse. Accordingly, the effective photosensitive area of the device was estimated to be ~50 μm×50 μm.

The responsivity was calculated using *R* = *I_ph_* / *P*, where *I_ph_* is the net photocurrent, defined as the difference between the current under illumination and the dark current, and *P* is the blackbody-radiation power incident on the effective photosensitive area of the device. The blackbody-radiation power was calculated using the Stefan-Boltzmann law and the corresponding radiometric relationships. In the calculation, the diameter of the blackbody aperture was 12.7 mm, and the distance between the blackbody aperture and the CBUP was 15 cm. The calculated blackbody-radiation power incident on the effective photosensitive area at different temperatures is shown in Figure S34c.

- **Section 15. Scanning photocurrent pattern under applied bias voltage**

**Figure S37. Scanning photocurrent pattern measured at an applied bias of 0.1 V.** Under bias, photocarriers in the channel are primarily drift-driven by the electric field, which suppresses the position-dependent bi-directional photoresponse. As a result, the scanning photocurrent pattern degrades into a map that reflects only the light intensity distribution. The scanning photocurrent pattern was obtained without using an external lock-in amplifier.

- **Section 16. Edge Extraction Performance Evaluation**

**Quantitative evaluation of the experimentally extracted edges**

To quantitatively evaluate the agreement between the experimentally extracted edges and the ideal edges, a binary edge map was generated from the measured scanning-photocurrent image in Figure 4c. The corresponding ground-truth edge map was constructed according to the known geometrical contours of the original letter pattern and its surrounding frame. Because an individual CBUP in the present experiment mainly extracts the local spatial gradient along its OEDV direction, only the target edges corresponding to this direction were retained in the ground truth.

The absolute value of the measured photocurrent was first taken to convert the positive and negative edge responses into a unified edge-magnitude representation. A fixed threshold of 1×10^-5^ A was then applied for binarization, with pixels below the threshold assigned a value of 0 and pixels equal to or above the threshold assigned a value of 1. The same threshold was maintained throughout the subsequent PFOM, SSIM, and localization-error calculations.

Pratt’s Figure of Merit (PFOM) was calculated as,

$$PFOM=\frac{1}{\max\left(N_{I},N_{D}\right)}\sum_{i=1}^{N_{D}} \frac{1}{1+\alpha d_{i}^{2}}$$

where *N_I_* and *N_D_* are the numbers of ideal-edge pixels and detected edge pixels, respectively, *d_i_* is the Euclidean distance from the *i*-th detected edge pixel to the nearest ideal-edge pixel, and *α* is the localization-error penalty factor. A commonly used value of *α* = 1/9 was adopted. The experimentally extracted CBUP edge map yields a PFOM of 0.63.

The mean edge localization error (MELE) was further calculated as the average distance from all detected edge pixels to their nearest ground-truth edge pixels. The resulting localization error is 5.54 pixels. This deviation may originate from several experimental factors, including the unequal positive and negative photoresponse weights of the two photosensitive regions, optical scattering at the mask edges, and edge broadening associated with photogenerated carriers in regions surrounding the channel. These factors can broaden or shift the experimentally measured edge response relative to the ideal geometrical boundary and thereby affect the localization of the binarized edge.

The Structural Similarity Index Measure (SSIM) between the experimental binary edge map and the ground truth was also calculated, giving a value of 0.86. SSIM is mainly designed to evaluate overall image similarity in terms of luminance, contrast, and structural information. For sparse binary edge maps dominated by background pixels, SSIM may be less sensitive to local edge displacement and a small number of false detections than PFOM and localization error. Therefore, SSIM is used here as a complementary metric, while PFOM and localization error are taken as the primary indicators of edge-extraction and localization performance.

**Operator-level benchmarking under unified conditions**

To avoid the influence of different input images, noise levels, and processing procedures on quantitative edge metrics, the OEDV, Sobel, and Canny methods were further compared numerically under identical input conditions. The same original image, ground-truth edge map, and artificial Gaussian-noise condition were used for all three methods. The input image was normalized to the range of 0-1, followed by the addition of zero-mean Gaussian noise with a standard deviation of *σ* = 0.03.

For the OEDV-based numerical analysis, two mutually orthogonal first-order difference vectors, [-1, 0, +1] and [-1, 0, +1]*^T^*, were used to obtain the *x-* and *y*-direction gradient responses, respectively, followed by gradient-magnitude fusion to generate a two-dimensional edge result. For the Sobel algorithm, the *x*- and *y*-direction gradients were similarly calculated and combined through the gradient magnitude. The Canny algorithm was implemented using its standard processing workflow, including smoothing, gradient calculation, non-maximum suppression (NMS), and thresholding, to obtain the final binary edge map. The original image, noisy image, and edge-extraction results obtained using the different methods are shown in Figure S38.

Under the same input and evaluation conditions, the OEDV-based numerical result gives PFOM = 0.89, SSIM = 0.90, and localization error = 0.98 pixels. The corresponding values for Sobel are PFOM = 0.90, SSIM = 0.91, and localization error = 0.94 pixels, while those for Canny are PFOM = 0.93, SSIM = 0.93, and localization error = 0.67 pixels. The OEDV-based numerical result therefore exhibits edge-extraction quality close to that of the Sobel operator under the same input and noise conditions, while Canny provides a lower localization error.

It should be emphasized that the OEDV results in this comparison are obtained from an idealized discrete numerical implementation and are intended to evaluate the edge-extraction capability of the spatial-differentiation mechanism represented by the OEDV. They do not represent the experimentally measured performance of the mechanically scanned CBUP. Practical devices are additionally affected by optical scattering, asymmetrical spatial response, carrier transport, and fabrication nonidealities. Furthermore, Sobel and particularly Canny are numerical image-processing methods with different processing workflows and computational complexity. The comparison therefore serves as an edge-quality benchmark under unified conditions rather than implying equivalent computational implementations.

**Figure S38. Numerical comparison of different edge-extraction methods under unified input conditions.** (a-i) Original image; (a-ii) zero-mean random Gaussian noise with *σ* = 0.03, with the image intensity normalized to the range of 0-1; (a-iii) input image after addition of Gaussian noise; (a-iv) ground-truth edge map. (b) Edge-extraction results based on two orthogonal OEDVs: (b-i) *x*-direction gradient; (b-ii) *y*-direction gradient; (b-iii) gradient magnitude; (b-iv) binary edge map. (c) Sobel-based edge-extraction results: (c-i) *x*-direction gradient; (c-ii) *y*-direction gradient; (c-iii) gradient magnitude; (c-iv) binary edge map. (d) Canny-based edge-extraction results, including intermediate results from the standard Canny workflow and the final binary edge map.

**Effective edge spatial resolution**

The effective edge spatial resolution of the current CBUP scanning system was further estimated from the experimentally measured spatial edge-response profile. The lateral photocurrent distribution extracted from Figure 4d crosses two adjacent edges of the outer frame of the target pattern and therefore contains two oppositely directed edge responses. After taking the absolute values of the positive and negative photocurrents, two localized edge-response peaks are obtained.

The experimentally measured edge-response peaks were interpolated and empirically fitted using Lorentzian functions to obtain smooth continuous edge-response profiles. The Lorentzian fitting is used only as an empirical representation of the measured response shape and does not imply that the physical edge response intrinsically follows a Lorentzian distribution. Figure S39a shows the experimentally measured responses of the two edges, the continuous data obtained by interpolation of the experimental results, and the corresponding fitting results.

To estimate the minimum resolvable separation between two adjacent edges, one fitted edge-response profile was fixed while the other was gradually shifted toward it (Figure S39b). The two edge-response curves were then superimposed using their experimentally corresponding amplitudes to obtain the combined responses at different edge separations, as shown in Figure S39c. A two-peak resolvability criterion was adopted: two edges were considered resolvable when the combined response retained two local maxima separated by a local minimum, whereas the edges were considered unresolved when the intermediate local minimum disappeared and the combined response became a single local peak.

**Figure S39. Analysis of the edge spatial response and two-edge resolvability of the CBUP.** (a) Position-dependent photocurrent profile extracted from Figure 4d, containing the responses to two adjacent edges, together with the experimental data, interpolated data, and empirical Lorentzian fitting results. (b) Simulated edge-response profiles at different edge separations obtained by varying the center-to-center distance between the two experimentally derived edge-response peaks. (c) Combined responses obtained by superimposing the two edge-response profiles at different edge separations, used to estimate the minimum resolvable edge separation according to the two-peak resolvability criterion.

According to this criterion, the two edge-response peaks remain just distinguishable at a separation of ~249 μm and merge into a single peak when the separation is further reduced. Therefore, the effective two-edge spatial resolution of the present CBUP scanning system is estimated to be ~249 μm.

The unequal amplitudes of the two edge-response peaks are consistent with the unequal positive and negative photoresponse weights of the experimental device. The value of approximately 249 μm represents an effective system-level spatial-resolution estimate under the present device dimensions, scanning step, optical configuration, and experimentally measured spatial-response profile. It should not be interpreted as a universal or intrinsic spatial-resolution limit of the CBUP.

- **Section 17. Photoresponse of the CBUP Array**

**Figure S40. Images of the CBUP array.** (a) Photograph of the CBUP array, with a surrounding ring of large electrodes used for wire bonding. (b) Schematic of the mask positioned above the array, showing that the transparent edge region does not fully cover the underlying device units. (c, d) Optical microscope images of the 10×10 CBUP array and a magnified view of the red-framed region. (e, f) Corresponding dark-field microscope images of panels (c) and (d). The horizontally flipped version of (d-f) is also reused in Figure 2.

Figure S40 shows images of a 10×10 CBUP array, in which the metal grating coverage of the left and right photosensitive regions in each unit is approximately 3:2, corresponding to an OEDV of [+1.0, 0, -1.2]. In simulations, due to the requirement of performing two-dimensional convolution, the edges of the simulated mask cannot span individual pixels in the same way as the actual mask edges. The experimental mask edges were aligned near the centers of the underlying unit devices to allow the CBUP to directly extract the target edges. In practical scenarios, if a target edge lies between two CBUPs, the output signal tends to weaken as the edge moves away from the photosensitive regions. Nevertheless, since most scenes are dynamic rather than static, slight movement of the CBUP or the optical lens can always ensure the target edges are captured.

According to Figure S41, both experimental and simulated results effectively extract the target edges. As shown in Figure S41c, the topmost left and right edges extracted in the simulation are located at horizontal coordinates 1.5 and 9.5, respectively, showing negative and positive signals. These results correspond well to the top edges of the simulated mask illustrated in Figure S41a.

In the experimental results shown in Figure S41e, the topmost left and right edges of the mask are located at unit positions 2 and 9 along the horizontal axis, with negative and positive photoresponse, respectively. These results also corresponding well to the top edges of the experimental mask in Figure S41b. Above results not only validate the edge-detection capability of the CBUP but also demonstrate the feasibility of using the photoresponse weight matrix to emulate a single CBUP unit. It is noteworthy that while two-dimensional convolution requires information from three pixels to detect edges, a single CBUP device can directly sense edge features.

For the readout requirements, in addition to a low-noise readout structure capable of detecting weak current signals, the readout circuit of a device array should preserve both the sign and magnitude of the current, rather than recording only its absolute value, because the CBUP generates positive and negative photocurrents associated with oppositely oriented edge features under zero external bias. Calibration of pixel-to-pixel variations in dark-state offset, photoresponse amplitude, and OEDV would also be required to reduce the influence of device nonuniformity on the determination of edge intensity and orientation. As the array size increases, pixel-by-pixel serial readout may become a limitation on the frame rate. Row-column addressing or column-parallel readout could therefore be adopted to enable the parallel addressing and readout of multiple pixels.

**Figure S41. Photocurrent patterns of the CBUP array.** (a, b) Schematic of the mask patterns used for simulation and experiment, where the red regions are transparent and the white regions are opaque. (c, e) Edge extraction results when the unit devices are arranged according to the OEDV of [+1.0, 0, -1.2], shown for simulation and experimental measurements, respectively. The blue dashed lines indicate the edge of the masks used in simulation and experiment. (d, f) Edge extraction results when the unit devices are arranged according to the transposed OEDV of [+1.0, 0, -1.2]^T^, shown for simulation and experimental measurements, respectively.

To evaluate device-to-device consistency, all 100 devices in the fabricated 10×10 array are characterized. The measured parameters included the dark current and the positive and negative photocurrents. The positive and negative photocurrents were measured with the centre of the focused optical spot positioned approximately over the photosensitive regions on opposite sides of each device, respectively, thereby representing oppositely oriented edge responses. The statistical distributions of these parameters are presented in Figure S42.

The response nonuniformity (*U*) was used to provide a preliminary evaluation of array uniformity and was defined as, *U* = *σ_I_* / |*μ_I_*| × 100%, where *σ_I_* is the standard deviation of the pixel responses and |*μ_I_*| is the mean response magnitude. The analysis shows that the standard deviation of the dark current across the array is 0.73 nA, while the nonuniformities of the positive and negative photocurrents are 0.53% and 0.55%, respectively. To further evaluate the relative consistency between the positive and negative edge outputs, we calculated the photocurrent-magnitude ratio (|*I*^-^| / |*I*^+^|) for each pixel. The nonuniformity of this ratio, calculated using the same definition, is 0.85%.

These results indicate that the devices within the present small-scale array exhibit relatively consistent responses. However, these statistical results apply only to the 10×10 array. As the array size increases, variations in electrical contacts, metallic patterns, Ge-film quality, and fabrication uniformity may increase device-to-device dispersion. Large-scale arrays will therefore require further fabrication optimization and pixel-level calibration to improve response uniformity.

**Figure S42 Statistical distributions of the electrical and photoresponse characteristics of the individual devices in the fabricated 10×10 array.** (a) Dark-current distribution. (b) Positive-photocurrent distribution. (c) Negative-photocurrent distribution. (d) Distribution of the negative-to-positive photocurrent-magnitude ratio.

Moreover, the contrast-to-noise ratio of the edge output of the 10×10 array is analysed. The edge and background regions were selected from the target-edge-device signals and non-edge-device signals (normalized output magnitude), respectively, in Figure S41e. The edge-output distinguishability was evaluated using the contrast-to-noise ratio (*CNR*), *CNR* = |*μ_edge_* - *μ_bg_*| /*σ_bg_*, where *μ_edge_* = 0.876 and *μ_bg_* = 0.0391 are the mean output magnitudes of the edge and background regions, respectively, and *σ_bg_* = 0.0549 is the standard deviation of the background-region output. This metric quantifies how many background standard deviations separate the mean edge output from the mean background output. The absolute values of the device output currents were used in the calculation to prevent the positive and negative edge polarities from cancelling each other. The resulting CNR is 15.3, which indicates that the edge output of the present small-scale array can be distinguished from the background.

Optical and thermal crosstalk are important issues that need to be evaluated in future large-scale array integration. Optical crosstalk may arise from diffraction, scattering, and lateral propagation of the incident light between adjacent pixels, causing unintended photoresponses in nontarget pixels and thereby reducing edge-localization accuracy and output contrast. In future array designs, this effect may be mitigated by optimizing the pixel pitch and imaging optics and by introducing metallic light-shielding regions or other optical-isolation structures between adjacent pixels.

For long-wave-infrared operation dominated by the photothermal mechanism, thermal crosstalk caused by the propagation of local temperature changes through the substrate or continuous material layers must also be considered. As the pixel pitch decreases, thermal crosstalk may increasingly limit the achievable integration density. This effect may be reduced by optimizing the lateral heat-conduction pathways between pixels or introducing suitable thermal-isolation structures. However, stronger thermal isolation may also affect the device response time and fabrication complexity. Future array development will therefore require a balance among thermal crosstalk, response speed, sensitivity, and pixel pitch.

- **Section 18. Photodetectors for On-chip Edge Extraction**

[33], [13], [34], [35], [36], [16], [15], [37], [38], [39], [40], [41]

**Table S4. Summary of Photodetectors for On-chip Edge Extraction**

Reconfigurable in-sensor computing photodetectors generally implement edge extraction by assigning positive, negative, or zero photoresponse weights to multiple neighboring devices or pixels and subsequently combining their outputs to perform convolution-like operations. In such schemes, electrical programming is commonly used to configure or update the response weights, while current or charge summation is required to obtain the final convolution output. This architecture provides high functional flexibility because the same array can, in principle, be reconfigured to implement different operators, such as Sobel or Laplacian kernels, and can support multiple image-preprocessing functions.

The CBUP follows a different physical-sensing strategy. Its spatial response weights are predefined by the device geometry and metallic-grating configuration, and the position-dependent bi-directional photoresponse directly produces a signed spatial-differentiation output along the OEDV direction within a single device. Therefore, the CBUP does not require electrical programming of multiple neighboring pixel weights or summation of their outputs to generate the edge response. The active region also operates without an externally applied bias. These characteristics can simplify the bias-control and interconnection requirements for dedicated edge sensing. In addition, a single CBUP is intrinsically sensitive to a local edge along its OEDV direction, whereas many convolution-based in-sensor computing schemes obtain an elementary edge response from the coordinated outputs of multiple neighboring pixels. However, this simplified fixed-function architecture also limits reconfigurability: once fabricated, the OEDV of the CBUP cannot be dynamically rewritten to implement arbitrary convolution kernels. Therefore, reconfigurable in-sensor computing devices are better suited to programmable and multifunctional image preprocessing, whereas the CBUP is more suitable for dedicated edge-sensing tasks in which the required spatial-differentiation function is predefined.

**References**

[1]	Tian, S., Yuan, W., Yu, Y., Guo, J., Liu, K., Tong, X., Chen, Q., Wu, Q., Quan, H., Zhou, J., et al., "Near-Infrared Polarization-Sensitive Detection by All-Si Plasmonic Hot Electron Detectors", *Nano Letters* **2024**, 24, 42, 13388 <https://doi.org/10.1021/acs.nanolett.4c03959>.

[2]	Zeng, L., Wu, D., Jie, J., Ren, X., Hu, X., Lau, S. P., Chai, Y., Tsang, Y. H., "Van der Waals Epitaxial Growth of Mosaic-Like 2D Platinum Ditelluride Layers for Room-Temperature Mid-Infrared Photodetection up to 10.6 microm", *Advanced Materials* **2020**, 32, 52, e2004412 <https://doi.org/10.1002/adma.202004412>.

[3]	Liu, W., Lv, J., Peng, L., Guo, H., Liu, C., Liu, Y., Li, W., Li, L., Liu, L., Wang, P., et al., "Graphene charge-injection photodetectors", *Nature Electronics* **2022**, 5, 5, 281 <https://doi.org/10.1038/s41928-022-00755-5>.

[4]	Xia, Z., Song, H., Kim, M., Zhou, M., Chang, T.-H., Liu, D., Yin, X., Xiong, K., Mi, H., Wang, X., et al., "Single-crystalline germanium nanomembrane photodetectors on foreign nanocavities", *Science Advances*, 3, 7, e1602783 <https://doi.org/10.1126/sciadv.1602783>.

[5]	Kim, C.-J., Lee, H.-S., Cho, Y.-J., Kang, K., Jo, M.-H., "Diameter-Dependent Internal Gain in Ohmic Ge Nanowire Photodetectors", *Nano Letters* **2010**, 10, 6, 2043 <https://doi.org/10.1021/nl100136b>.

[6]	Hu, W., Cong, H., Huang, W., Huang, Y., Chen, L., Pan, A., Xue, C., "Germanium/perovskite heterostructure for high-performance and broadband photodetector from visible to infrared telecommunication band", *Light: Science & Applications* **2019**, 8, 1, 106 <https://doi.org/10.1038/s41377-019-0218-y>.

[7]	Going, R. W., Loo, J., Liu, T. J. K., Wu, M. C., "Germanium Gate PhotoMOSFET Integrated to Silicon Photonics", *IEEE Journal of Selected Topics in Quantum Electronics* **2014**, 20, 4, 1 <https://doi.org/10.1109/JSTQE.2013.2294470>.

[8]	Jiang, H., Wei, J., Sun, F., Nie, C., Fu, J., Shi, H., Sun, J., Wei, X., Qiu, C. W., "Enhanced Photogating Effect in Graphene Photodetectors via Potential Fluctuation Engineering", *ACS Nano* **2022**, 16, 3, 4458 <https://doi.org/10.1021/acsnano.1c10795>.

[9]	Mueller, T., Xia, F., Avouris, P., "Graphene photodetectors for high-speed optical communications", *Nature Photonics* **2010**, 4, 5, 297 <https://doi.org/10.1038/nphoton.2010.40>.

[10]	Li, X., Wu, J., Mao, N., Zhang, J., Lei, Z., Liu, Z., Xu, H., "A self-powered graphene–MoS2 hybrid phototransistor with fast response rate and high on–off ratio", *Carbon* **2015**, 92, 126 <https://doi.org/https://doi.org/10.1016/j.carbon.2015.03.064>.

[11]	Peng, M., Xie, R., Wang, Z., Wang, P., Wang, F., Ge, H., Wang, Y., Zhong, F., Wu, P., Ye, J., et al., "Blackbody-sensitive room-temperature infrared photodetectors based on low-dimensional tellurium grown by chemical vapor deposition", *Science Advances* **2021**, 7, 16: eabf7358 <https://doi.org/10.1126/sciadv.abf7358>.

[12]	Peng, Y., Yang, Q., Fu, J., Yang, J., Wu, Y., Zhang, Y., Li, G., Li, Z., Shi, H., Wei, X., "Uncooled Graphene Infrared Detectors Enabled by Pyroelectric Photogating for Human Radiation Perception", *Laser & Photonics Reviews* **2025**, 19, 17, 2500218 <https://doi.org/https://doi.org/10.1002/lpor.202500218>.

[13]	Fu, J., Nie, C., Sun, F., Li, G., Shi, H., Wei, X., "Bionic visual-audio photodetectors with in-sensor perception and preprocessing", *Science Advances* **2024**, 10, 7, eadk8199 <https://doi.org/10.1126/sciadv.adk8199>.

[14]	Wang, C.-Y., Liang, S.-J., Wang, S., Wang, P., Li, Z. a., Wang, Z., Gao, A., Pan, C., Liu, C., Liu, J., et al., "Gate-tunable van der Waals heterostructure for reconfigurable neural network vision sensor", *Science Advances* **2020**, 6, 26: eaba6173 <https://doi.org/10.1126/sciadv.aba6173>.

[15]	Lee, S., Peng, R., Wu, C., Li, M., "Programmable black phosphorus image sensor for broadband optoelectronic edge computing", *Nature Communications* **2022**, 13, 1, 1485 <https://doi.org/10.1038/s41467-022-29171-1>.

[16]	Tan, F., Cao, Y., Sang, W., Han, Z., Li, H., Wang, T., Songlu, W., Gan, Y., Yu, Y., Zhang, X., et al., "Multifunctional broadband artificial visual system using all-in-one two-dimensional optoelectronic transistors", *Materials Today* **2024**, 81, 23 <https://doi.org/https://doi.org/10.1016/j.mattod.2024.10.003>.

[17]	Fu, J., Ji, L., Wu, Z., Li, G., Nie, C., Xiong, W., Wang, F., Sun, F., Zhou, Y., Zang, Z., et al., "An all-in-one optoelectronic logic device with self-distinguishable dual-band photoresponse", *Device* **2024**, 2, 4, 100321 <https://doi.org/https://doi.org/10.1016/j.device.2024.100321>.

[18]	Ni, Z., Ma, L., Du, S., Xu, Y., Yuan, M., Fang, H., Wang, Z., Xu, M., Li, D., Yang, J., et al., "Plasmonic Silicon Quantum Dots Enabled High-Sensitivity Ultrabroadband Photodetection of Graphene-Based Hybrid Phototransistors", *ACS Nano* **2017**, 11, 10, 9854 <https://doi.org/10.1021/acsnano.7b03569>.

[19]	Luo, P., Zhuge, F., Wang, F., Lian, L., Liu, K., Zhang, J., Zhai, T., "PbSe Quantum Dots Sensitized High-Mobility Bi2O2Se Nanosheets for High-Performance and Broadband Photodetection Beyond 2 mum", *ACS Nano* **2019**, 13, 8, 9028 <https://doi.org/10.1021/acsnano.9b03124>.

[20]	Jiang, H., Wang, M., Fu, J., Li, Z., Shaikh, M. S., Li, Y., Nie, C., Sun, F., Tang, L., Yang, J., et al., "Ultrahigh Photogain Short-Wave Infrared Detectors Enabled by Integrating Graphene and Hyperdoped Silicon", *ACS Nano* **2022**, 16, 8, 12777 <https://doi.org/10.1021/acsnano.2c04704>.

[21]	Jiang, H., Fu, J., Wei, J., Li, S., Nie, C., Sun, F., Wu, Q. Y. S., Liu, M., Dong, Z., Wei, X., et al., "Synergistic-potential engineering enables high-efficiency graphene photodetectors for near- to mid-infrared light", *Nature Communications* **2024**, 15, 1, 1225 <https://doi.org/10.1038/s41467-024-45498-3>.

[22]	Wei, J., Xu, C., Dong, B., Qiu, C.-W., Lee, C., "Mid-infrared semimetal polarization detectors with configurable polarity transition", *Nature Photonics* **2021**, 15, 8, 614 <https://doi.org/10.1038/s41566-021-00819-6>.

[23]	Adinolfi, V., Sargent, E. H., "Photovoltage field-effect transistors", *Nature* **2017**, 542, 7641, 324 <https://doi.org/10.1038/nature21050>.

[24]	Fu, J., Leng, C., Ma, R., Nie, C., Sun, F., Li, G., Wei, X., "Photo-driven fin field-effect transistors", *Opto-Electronic Science* **2024**, 3, 5, 230046 <https://doi.org/10.29026/oes.2024.230046>.

[25]	Kimata, M., Denda, M., Iwade, S., Yutani, N., Tsubouchi, N., "A wide spectral band photodetector with PtSi/p-Si Schottky-barrier", *International Journal of Infrared and Millimeter Waves* **1985**, 6, 10, 1031 <https://doi.org/10.1007/BF01010679>.

[26]	Shoda, M., Akagawa, K., Kazama, T. 1996, *410K pixel PtSi Schottky-barrier infrared CCD image sensor*. SPIE.

[27]	Elabd, H., Villani, T., Tower, J., "High density Schottky barrier IRCCD sensors for SWIR applications at intermediate temperature", *NASA. Goddard Space Flight Center The Multispectral Imaging Science Working Group, Vol. 3* **1982**.

[28]	Tsaur, B. Y., Chen, C. K., Nechay, B. A., "IrSi Schottky-barrier infrared detectors with wavelength response beyond 12 mu m", *IEEE Electron Device Letters* **1990**, 11, 9, 415 <https://doi.org/10.1109/55.62974>.

[29]	Czernik, A., Palm, H., Cabanski, W., Schulz, M., Suckow, U., "Infrared photoemission of holes from ultrathin (3–20 nm) Pt/Ir-compound silicide films into silicon", *Applied Physics A* **1992**, 55, 2, 180 <https://doi.org/10.1007/BF00334221>.

[30]	Casalino, M., Russo, R., Russo, C., Ciajolo, A., Di Gennaro, E., Iodice, M., Coppola, G., "Free-Space Schottky Graphene/Silicon Photodetectors Operating at 2 um", *ACS Photonics* **2018**, 5, 11, 4577 <https://doi.org/10.1021/acsphotonics.8b01037>.

[31]	Knight, M. W., Sobhani, H., Nordlander, P., Halas, N. J., "Photodetection with Active Optical Antennas", *Science* **2011**, 332, 6030, 702 <https://doi.org/10.1126/science.1203056>.

[32]	Desiatov, B., Goykhman, I., Mazurski, N., Shappir, J., Khurgin, J. B., Levy, U., "Plasmonic enhanced silicon pyramids for internal photoemission Schottky detectors in the near-infrared regime", *Optica* **2015**, 2, 4, 335 <https://doi.org/10.1364/OPTICA.2.000335>.

[33]	Wu, L., Shi, M., Dai, Z., Ye, T., Deng, J., Yu, Y., Wang, R., Bu, Y., Cui, T., Ma, J., et al., "Visible-to-Mid-Infrared In-Sensor Computing With a Reconfigurable Black Phosphorus Photodiode", *IEEE Electron Device Letters* **2024**, 45, 7, 1217 <https://doi.org/10.1109/LED.2024.3403934>.

[34]	Jang, H., Hinton, H., Jung, W.-B., Lee, M.-H., Kim, C., Park, M., Lee, S.-K., Park, S., Ham, D., "In-sensor optoelectronic computing using electrostatically doped silicon", *Nature Electronics* **2022**, 5, 8, 519 <https://doi.org/10.1038/s41928-022-00819-6>.

[35]	Pi, L., Wang, P., Liang, S.-J., Luo, P., Wang, H., Li, D., Li, Z., Chen, P., Zhou, X., Miao, F., et al., "Broadband convolutional processing using band-alignment-tunable heterostructures", *Nature Electronics* **2022**, 5, 4, 248 <https://doi.org/10.1038/s41928-022-00747-5>.

[36]	Zhang, Z., Wang, S., Liu, C., Xie, R., Hu, W., Zhou, P., "All-in-one two-dimensional retinomorphic hardware device for motion detection and recognition", *Nature Nanotechnology* **2022**, 17, 1, 27 <https://doi.org/10.1038/s41565-021-01003-1>.

[37]	Han, C., Liu, X., Han, X., He, M., Han, J., Zhang, H., Hou, X., Zhou, H., Yu, H., Wu, Z., et al., "High-Performance Phototransistor Based on Graphene/Organic Heterostructure for In-Chip Visual Processing and Pulse Monitoring", *Advanced Functional Materials* **2022**, 32, 52, 2209680 <https://doi.org/https://doi.org/10.1002/adfm.202209680>.

[38]	Zhu, Y., Wang, Y., Pang, X., Jiang, Y., Liu, X., Li, Q., Wang, Z., Liu, C., Hu, W., Zhou, P., "Non-volatile 2D MoS2/black phosphorus heterojunction photodiodes in the near- to mid-infrared region", *Nature Communications* **2024**, 15, 1, 6015 <https://doi.org/10.1038/s41467-024-50353-6>.

[39]	Zhang, Q., Zhang, Y., Liu, X., Zhang, N., Miao, T., Hu, H., Wang, L., Zhu, Z., "Ge-Based Visible-Infrared Bipolar Floating-Gate Phototransistor for Broad-Spectrum Retinal Bionics", *Advanced Science* **2025**, 12, 45, e12649 <https://doi.org/https://doi.org/10.1002/advs.202512649>.

[40]	Wu, G., Zhang, X., Feng, G., Wang, J., Zhou, K., Zeng, J., Dong, D., Zhu, F., Yang, C., Zhao, X., et al., "Ferroelectric-defined reconfigurable homojunctions for in-memory sensing and computing", *Nature Materials* **2023**, 22, 12, 1499 <https://doi.org/10.1038/s41563-023-01676-0>.

[41]	Zhang, S., Xiao, P., Hong, Q., Tang, L., Xie, Z., He, R., Jiang, B., Hong, X., Li, X., Zhu, H., et al., "Rational design of a vision fusion system with visible and near-infrared spectral integration for improved environmental perception", *National Science Review* **2025**, 12, 7, nwaf204 <https://doi.org/10.1093/nsr/nwaf204>.
